# Evaluation of conditional cash transfers and mHealth audio messaging in reduction of risk factors for childhood malnutrition in internally displaced persons camps in Somalia: A 2 × 2 factorial cluster-randomised controlled trial

**DOI:** 10.1371/journal.pmed.1004180

**Published:** 2023-02-27

**Authors:** Carlos S. Grijalva-Eternod, Mohamed Jelle, Hani Mohamed, Katie Waller, Bishar Osman Hussein, Emmanuel Barasa, Andrea Solomon, Sajia Mehjabeen, Andrew Copas, Edward Fottrell, Andrew J. Seal

**Affiliations:** 1 UCL Institute for Global Health, London, United Kingdom; 2 London School of Hygiene and Tropical Medicine, London, United Kingdom; 3 Concern Worldwide, Mogadishu, Somalia; 4 Concern Worldwide US, New York, United States of America; 5 Concern Worldwide, Dublin, Ireland; The Hospital for Sick Children, CANADA

## Abstract

**Background:**

Cash transfer programmes are increasingly used in humanitarian contexts to help address people’s needs across multiple sectors. However, their impact on the key objectives of reducing malnutrition and excess mortality remains unclear. mHealth interventions show great promise in many areas of public health, but evidence for their impact on reducing the risk factors for malnutrition is uncertain. We therefore implemented a trial to determine the impacts of 2 interventions in a protracted humanitarian context, a cash transfer conditionality and mHealth audio messages.

**Methods and findings:**

A 2 × 2 factorial cluster-randomised trial was implemented in camps for internally displaced people (IDP) near Mogadishu, Somalia, starting in January 2019. The main study outcomes were assessed at midline and endline and included coverage of measles vaccination and the pentavalent immunisation series, timely vaccination, caregiver’s health knowledge, and child diet diversity. Twenty-three clusters (camps) were randomised to receive or not receive conditional cash transfers (CCTs) and an mHealth intervention, and 1,430 households were followed up over 9 months. All camps received cash transfers made at emergency humanitarian level (US$70/household/month) for 3 months followed by a further 6 months at a safety net level (US$35). To be eligible to receive cash, households in camps receiving CCT were required to take their children <5 years age to attend a single health screening at a local clinic and were issued with a home-based child health record card. Participants in camps receiving the mHealth intervention were asked (but not required) to listen to a series of audio messages about health and nutrition that were broadcast to their mobile phone twice a week for 9 months. Participants and investigators were not blinded. Adherence to both interventions was monitored monthly and found to be high (>85%). We conducted intention-to-treat analysis.

During the humanitarian intervention phase, the CCT improved coverage of measles vaccination (MCV1) from 39.2% to 77.5% (aOR 11.7, 95% CI [5.2, 26.1]; *p* < 0.001) and completion of the pentavalent series from 44.2% to 77.5% (aOR 8.9, 95% CI [2.6, 29.8]; *p* = < 0.001). By the end of the safety net phase, coverage remained elevated from baseline at 82.2% and 86.8%, respectively (aOR 28.2, 95% CI [13.9, 57.0]; *p* < 0.001 and aOR 33.8, 95% CI [11.0, 103.4]; *p* < 0.001). However, adherence to timely vaccination did not improve. There was no change in the incidence of mortality, acute malnutrition, diarrhoea, or measles infection over the 9 months of follow-up.

Although there was no evidence that mHealth increased Mother’s knowledge score (aOR 1.32, 95% CI [0.25, 7.11]; *p* = 0.746) household dietary diversity increased from a mean of 7.0 to 9.4 (aOR 3.75, 95% CI [2.04, 6.88]; *p* < 0.001). However, this was not reflected by a significant increase in child diet diversity score, which changed from 3.19 to 3.63 (aOR 2.1, 95% CI [1.0, 4.6]; *p* = 0.05). The intervention did not improve measles vaccination, pentavalent series completion, or timely vaccination, and there was no change in the incidence of acute malnutrition, diarrhoea, measles infection, exclusive breastfeeding, or child mortality. No significant interactions between the interventions were found. Study limitations included the limited time available to develop and test the mHealth audio messages and the necessity to conduct multiple statistical tests due to the complexity of the study design.

**Conclusions:**

A carefully designed conditionality can help achieve important public health benefits in humanitarian cash transfer programmes by substantially increasing the uptake of child vaccination services and, potentially, other life-saving interventions. While mHealth audio messages increased household diet diversity, they failed to achieve any reductions in child morbidity, malnutrition, or mortality.

**Trial registration:**

ISRCTN ISRCTN24757827. Registered November 5, 2018.

## Abstract 2

In a 2x2 factorial cluster-randomised controlled trial, Andrew Seal and colleagues evaluate the impact of conditional cash transfers and mHealth audio messaging on childhood malnutrition in internally displaced persons camps in Somalia.

## Author summary

**Why was this study done?:**

- The use of cash transfers in humanitarian emergencies is commonplace, but there is limited information on how they impact child mortality or acute malnutrition.
- Conditional cash transfers are not frequently used in humanitarian programming, despite their ability to promote healthy behaviours.
- mHealth, or mobile health, is a term used for the practice of medicine and public health supported by mobile devices. The use of complementary interventions, such as mHealth behaviour change communication, may help to increase the impact of cash transfers on health and nutrition outcomes.

**What did the researchers do and find?:**

- During 2019, we conducted a 9-month, cluster-randomised controlled trial, in camps for displaced people near to Mogadishu in Somalia.
- Using a 2 × 2 factorial design, we studied the use of a cash conditionality and mHealth audio messages to improve health-seeking behaviour and reduce risk factors for malnutrition.
- The conditionality involved a one time point health screening, and 2 audio messages were broadcast to participant’s phones, each week for 8 months.
- Cash conditionality significantly improved the coverage of measles and pentavalent vaccination, although timely vaccination for all antigens did not improve.
- The mHealth intervention did not improve any of the primary trial outcomes, although it was associated with a higher household diet diversity.

**What do these findings mean?:**

- mHealth audio messages were well accepted, and further work to refine and iteratively test them may be useful.
- A carefully designed, simple, cash conditionality can substantially improve coverage of key public health interventions and help reduce the risk factors for malnutrition and excess mortality in a humanitarian context.
- Further work to scale up and test cash conditionality in different contexts is warranted.

## Introduction

In low- and middle-income countries, acute malnutrition affects 45 million children aged <5 years [1]. This is a serious global health concern as acute malnutrition is a leading cause of death in children, accounting for 11.5% of total deaths, and contributes significantly to the overall disease burden [2]. Populations affected by natural disasters or conflicts often suffer from a greater prevalence of acute malnutrition, and infectious disease is one of its immediate causes [3]. Each year immunization averts an estimated 2.5 million deaths among children under 5 years old and a much larger number of infections, and this also contributes to controlling malnutrition. However, weak health systems are a major constraint on the effectiveness of immunization programmes, and these challenges may be exacerbated in fragile states and humanitarian contexts [4,5].

Cash-based interventions (CBIs) are increasingly used as a humanitarian response to emergencies and have gained greater acceptance compared with in-kind interventions [6]. Among the reasons behind this greater acceptance is the perception that CBI increases beneficiary agency, as they can often decide how to use the cash for their specific needs of goods or services; it is a cost-effective intervention, has positive impacts on local economies, and improves beneficiary satisfaction. However, CBIs do not appear to consistently reduce the risk of malnutrition in children, and there is no evidence on its ability to reduce excess mortality [6–10]. Even in nonhumanitarian contexts debate continues on the relative merits of conditional and unconditional transfers on child health and nutrition and how that may play out in different contexts [11,12].

The prevalence of acute child malnutrition in Somalia is among the highest in the world as a result of the political instability and conflict afflicting the country since 1991, together with recurrent natural disasters such as droughts [3,13,14]. Because of this protracted state of conflict, regional agriculture and trade is disrupted, which exacerbates food insecurity by increasing food prices, especially in urban areas [9]. Conflict also causes forced displacement and reduces humanitarian access, hinders the provision of health services, and increases the risk of mortality. In 2017 to 2018, acute malnutrition affected 1.2 million children in Somalia, of which over 230,000 were severely malnourished [15]. Internally displaced people (IDP) are the population group most affected by acute malnutrition and disease outbreaks, as they often live in peri-urban camps that lack adequate access to essential services. Studies have also indicated that low knowledge of health and nutrition issues may contribute to the risk of malnutrition [16].

CBIs were first implemented at scale in Somalia in 2011 to respond to the famine crisis and were seen as an essential strategy for providing food assistance, given the existence of functioning markets and the limited humanitarian access to southern Somalia [17]. Recently, as part of the Research on Food Assistance for Nutritional Impact (REFANI) consortium, Concern Worldwide and University College London (UCL) tested if an emergency multipurpose cash transfer, implemented over a 5-month period, would reduce acute malnutrition risk in children aged <5 years living in IDP camps in the Afgooye Corridor, Mogadishu [18]. The CBI in the REFANI-S study failed to reduce the incidence of acute malnutrition despite significantly improving food security, measured at the household and individual level [7]. This finding suggested the need for modification of the CBI by adding elements such as behaviour change communications (BCCs), which have been shown to reduce acute malnutrition risk in some contexts [19].

mHealth, or mobile health, is a term used for the practice of medicine and public health supported by mobile devices. The Cash for Improved Nutrition in Somalia (CINS) study aimed to assess whether modifying a CBI, by adding a one-time conditionality of health screening children at the local health clinic, or a mHealth BCC intervention delivered through mobile phones, could reduce the risk factors for acute malnutrition in children, aged 0 to 59 months, living in IDP camps. We tested 2 hypotheses. Firstly, that the conditional cash intervention would improve vaccination coverage, and secondly, that the mHealth intervention would increase mother/caregiver’s health and nutrition knowledge, and improve caring behaviours, as indicated by improved vaccination coverage and a greater child diet diversity score (IDDS).

## Methods

### Ethical approvals and trial registration

The Ministry of Health & Human Services of the Federal Republic of Somalia (reference: MOH&HS/DGO/0993/Jun/2018) and the Research Ethics Committee of UCL (project ID: 4684/001) granted ethical approval. The study was registered on November 5, 2018, with ISRCTN (ISRCTN24757827). This study is reported as per the Consolidated Standards of Reporting Trials (CONSORT) guideline (S1 CONSORT Checklist). A consort checklist for this paper is included in the Supporting information (S1 CONSORT Checklist).

### Setting

The trial took place in the Afgooye Corridor, in peri-urban Mogadishu, Somalia. The Afgooye Corridor hosts the largest IDP camps in Mogadishu, the cumulative result of multiple protracted crises in the country over the years. The IDP camps are spontaneous and privately run settlements, often managed by informal settlement managers. The camps are often overcrowded, lack basic sanitation and health services, and residents face recurrent evictions. The IDP living in these camps subsist mostly on humanitarian assistance from relief agencies and from casual labour when available.

### Interventions

All study participants were enrolled in a cash transfer programme and received the same level of transfers. We tested 2 separate interventions that were delivered as part of this cash transfer programme: (1) a cash transfer conditionality, where household registration for the cash transfer programme, was conditional on a one-time health screening of their children aged 0 to 59 months; and (2) an mHealth BCC intervention, where we transmitted prerecorded audio messages to the mobile phones of people registered for the cash transfer programme.

### Cash transfer programme

The cash transfer programme consisted of 3 monthly electronic transfers of US$70.00 (henceforth humanitarian cash transfer), followed by 6 monthly electronic transfers of US$35.00 (henceforth safety net cash transfer). The humanitarian cash was aligned to the recommended transfer value from the Somali Cash Working Group, which reflected 80% of the multipurpose (i.e., full) minimum expenditure basket (MEB) for the given location. The safety net cash transfer value followed standard practice of the implementing agency at the time of intervention design, i.e., half the full humanitarian value. Whereas the higher value was distributed at times of peak hunger, the safety net value was lower so as to reach as many households in need as possible while supporting household income, promote positive coping strategies, and reduce risk of dependence.

Upon registration, a household female representative received a mobile phone SIM card with a unique number, which was recorded and through which they received the cash transfer via a mobile money transfer made by the telecommunications company we contracted (Hormuud Telecom Somalia). The cash transfer programme targeted women as the household cash recipient on the assumption that their spending was more likely to benefit their children. Cash transfers were made on March 24, April 27, June 3, July 4, July 27, August 25, September 29, October 27, and December 8, 2019.

### Cash transfer conditionality

The conditionality intervention, henceforth the conditional cash transfer (CCT) intervention, was a one-time requirement for all children aged 0 to 59 months to attend the local clinic at the beginning of the study, where health staff screened their health and issued them with a CINS health record card. The child was offered any necessary vaccinations, but agreement to receive vaccination was not necessary to meet the conditionality. Children born after registration for the cash transfer had taken place were not required to attend.

Households in the CCT intervention arm were actively encouraged to meet the conditionality, which all did. If households had failed to meet the conditionality, they would have been denied registration for the cash transfer programme. During registration for the cash transfer programme, we verified whether households in the CCT arm met the conditionality by asking the mothers/caregiver to show the child CINS health card. Additional checks of the child’s CINS health card were carried out monthly by study staff and community health workers (CHWs) to encourage any newly arrived children, in the households of the CCT arm, to be health screened and receive their CINS health card. The health record card was designed for the study based on recommendations from the World Health Organisation (WHO), the existing MoH Child Health booklet, and experience in Malawi, where passport-sized, home-based health record cards had been developed [21]. The card was designed to be able to fit in a pocket and had a water-resistant cover. It received some limited pretesting with volunteer Concern staff before being printed in Mogadishu. Study health record cards were only issued to children living in the CCT intervention arm camps.

All households in the CCT and unconditional cash transfer (UCT) arms had similar access to the local health clinic. In addition, CHWs visited all households in all the study arms each month to review the vaccination status of their children, aged 0 to 59 months, and to remind their caregivers of any due child vaccinations.

### mHealth behaviour change communication

The mHealth intervention consisted of a series of 30 pairs of health-promoting audio messages. Each pair of messages comprised a 2-minute long drama and a 1-minute long reenforcing message that were transmitted on separate days, usually on Friday for the drama and Sunday for the reenforcement, to the recipient’s mobile phone. The message series comprised 5 seasons, each of 6 pairs of messages and transmission of the whole series took place over 8 months (May to December 2019). The drama component was a serialised fictional story, following a family similar to those in the study camps. The content of the message series covered 6 topics: (1) vaccination; (2) water, sanitation, and hygiene (WASH); (3) infant and young child feeding (IYCF) practices; (4) identifying signs of serious illness and seeking care; (5) recognition, treatment, and prevention of acute malnutrition; and (6) maximizing health and nutrition for all household members. We identified these 6 topics as important barriers to optimal health and nutrition during the implementation of the REFANI-S study. [7] We designed the message content based on information gathered during a formative human-centred design workshop held in Mogadishu with Concern staff, CHWs, and mothers and fathers from the IDP community. The design process was also informed by the theoretical behaviour change approach used in a previous mHealth project in Bangladesh [22]. We developed the messages in collaboration with 2 Somali companies that specialised in interactive voice response (IVR) technology (Shaqodoon Organisation, Hargeisa, Somaliland) and creative message development (Media INK, Mogadishu, Somalia). Messages were recorded in Mahrati and Maay, the 2 Somali languages widely spoken in the IDP camps, and recipients received broadcasts in their preferred language.

### Trial design and participants

We implemented a 2 × 2 factorial cluster-randomised controlled trial, where IDP camps were the units of randomisation. We surveyed 23 clusters between January 17 and February 12, 2019. We used 3 criteria to select these clusters: first, the clusters should not be receiving any cash assistance from another organisation; second, the clusters needed to be located between 0.3 and 1 km straight-line distance from the Concern Worldwide–operated health centre, where we planned to undertake the health screening of children; and third, clusters needed to be of comparable size. There were no exclusion criteria for individuals participating in the study except that the household was required to have a child aged 0 to 59 months.

Informed verbal consent was obtained from camp leaders in all IDP camps before starting data collection. In addition, following a detailed explanation of the study objectives and data collection process, informed verbal and written consent was obtained from caregivers at the household. Study participants were informed about their right to withdraw from the study and that participation or withdrawal from the study would not affect their entitlement to humanitarian assistance. Confidentiality and data security of the respondents was ensured by the use of password-protected encrypted devices and the removal of any information from datasets that could be used to identify respondents or their location.

**Fig 1:**
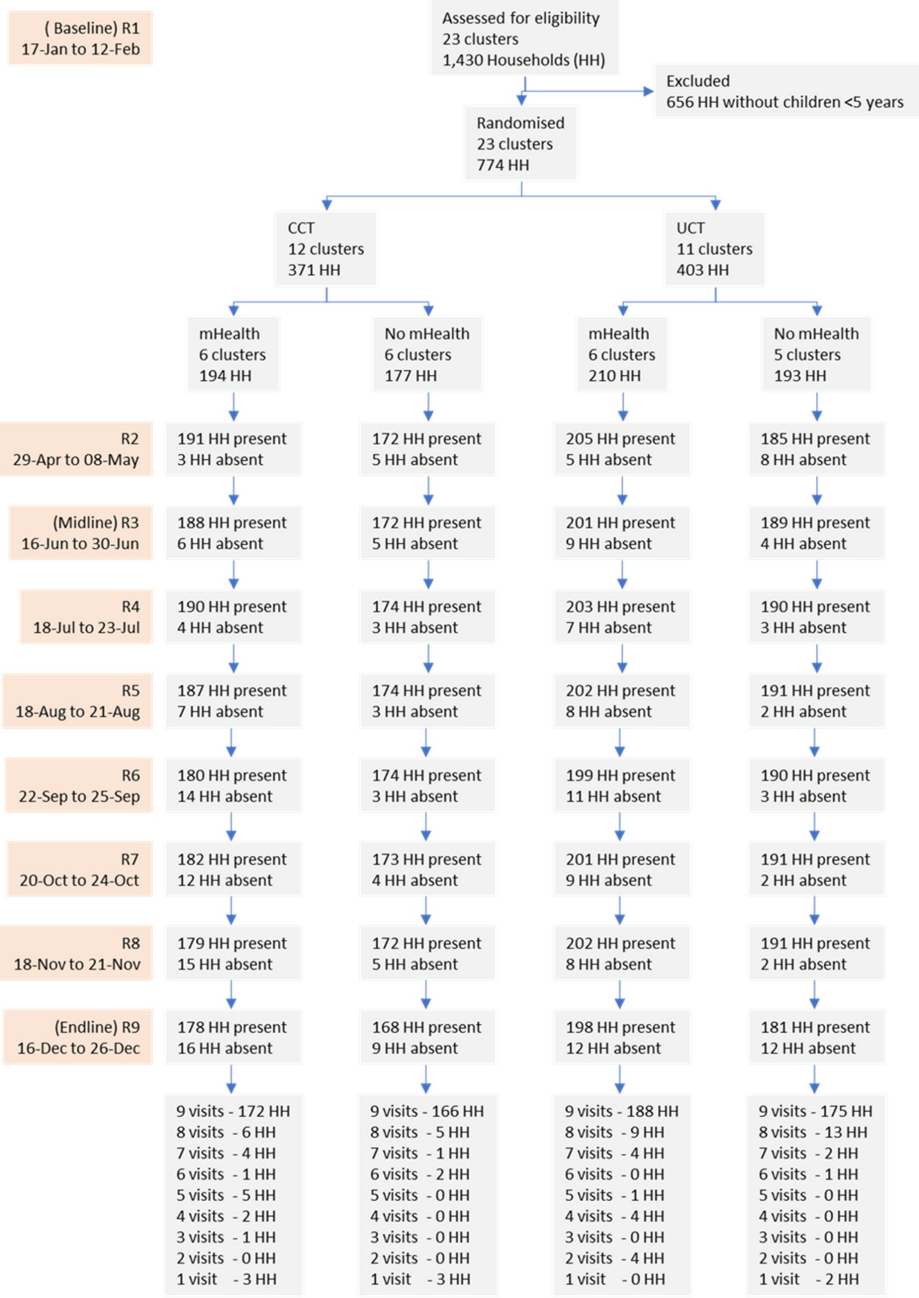
Study flow chart for households. CCT, conditional cash transfer; HH, household; R, data collection round; UCT, unconditional cash transfer.

We planned to include 20 clusters in the study with an average of 65 children, aged 0 to 59 months, per cluster. However, the surveyed clusters had fewer children than expected (average 54.3 children, range: 11 to 102), so we included all 23 surveyed clusters in the study, and we used the Round 1 (R1) data that we collected as our baseline. We then randomly allocated half the 23 clusters to the CCT intervention resulting in 12 clusters assigned to this intervention and 11 to the control (see **Fig 1**). The allocation was done by first listing the clusters in order of size (number of households) and then randomly assigning the CCT intervention or control to alternate members of the list, using a random number, generated using the Excel RANDBETWEEN() function, to determine the allocation for the first cluster. The process was then repeated in an identical way to randomly allocate the mHealth intervention, separately, to the CCT control and intervention groups. The randomisation of interventions was performed in London by the study PI. As with most interventions of this nature, participants could not be “blinded” to allocation status. Within each cluster, all households with children aged <5 years (*n =* 774) were eligible to be included in the study, together with all the children aged <5 years and their mothers/caregivers that were members of these households.

Following the baseline survey and cluster randomisation, the cash distribution and mHealth interventions started. We then implemented 8 additional rounds of data collection from the study households, beginning on April 29, 2019 (see **Fig 1**). The “midline” data collection took place at R3 in June (3 months after the start of the intervention), and the “endline” took place at R9 in December (9 months after the start of the intervention). In all 9 rounds of data collection, the sample of households and mothers/caregivers remained the same (a closed household cohort). However, we included in the study all the children aged <5 years that joined the household after baseline.

We collected information at the household and individual level, i.e., mother/caregiver and child (see S1 **Table**). We gathered data from household heads and mothers/primary carers regarding household demographics, food security, expenditure, morbidity, WASH, breastfeeding practices, mother/caregiver’s knowledge on health, and nutrition and vaccination programme coverage. We also collected mid-upper arm circumference (MUAC) measurements and assessed bipedal pitting oedema (henceforth oedema). We identified children as acutely malnourished if they presented with a MUAC <12.5 cm and/or oedema and referred them to the health centre for treatment. We identified all children <5 years old who died between R1 and R9 data collections and arranged a verbal autopsy (VA) interview with their mothers/caregivers.

### Field procedures and data handling

Data were collected by the research field teams using a structured questionnaire, translated into Somali, on mobile Android devices running Open Data Kit (ODK). Data collection at baseline (month 0), midline (after 3 months of intervention), and endline (after 9 months of intervention) was conducted by study enumerators, who were hired specifically for the data collection exercises. Data were collected at the monthly time points in between 0, 3, and 9 months by CHWs. Prior to starting data collection, we implemented 1-week training sessions for enumerators and supervisors at R1, R3, and R9 data collection rounds. During these training sessions, we piloted and revised questionnaires. The R1, R2, and R3 data collection was undertaken by 10 teams. Each team had an enumerator and 2 CHWs. The rest of data collection rounds were conducted by 10 teams of CHWs, with each team having 2 members. Teams in all rounds were supervised by 4 nutrition supervisors, under the overall supervision of a field study coordinator. During each round of data collection, if we could not reach a household after repeated attempts or any of its members were absent, we enquired about the reasons for this absence from family or neighbours.

### Household demographics

To understand household composition, we collected data on the numbers of members and their ages. We then estimated total, child, and aged dependency ratios as the ratio between the number of members aged <15 years or aged ≥65 years, members aged <15 years, and members aged ≥65 years, each divided by the number of members aged 15 to 64 years, respectively. Additionally, we obtained information on the sex of the head of household, and whether they lived in a monogamous or polygamous arrangement.

### Water, sanitation, and hygiene

Using a questionnaire and through observation, we obtained data on whether households had access to piped water, whether they pay for this access, and the time that it took them to collect water. We also obtained data on household access to handwashing facilities, soap availability, and the type of toilet. We classified households as engaging in open defecation if their members reported to relieve themselves in open fields or if their household showed no evidence, reported or observed, of having access to latrines.

### Food security

We used household and child dietary diversity score (DDS) and the reduced coping strategies index (rCSI) to assess food security. We applied a 24-hour food recall to a checklist of 12 food groups for estimating the household DDS, as recommended by the Food and Agriculture Organisation of the United Nations [23]. The household DDS food groups were as follows: (1) cereals; (2) white tubers and roots; (3) vegetables; (4) fruits; (5) meat; (6) eggs; (7) fish and seafood; (8) pulses, nuts, and seeds; (9) dairy products; (10) oils and fats; (11) sweets; and (12) spices, condiments, and beverages. The household DDS has a range of 0 to 12. Similarly, we applied a 24-hour food recall to a checklist of 7 food groups to estimate child DDS, as recommended by WHO [24]. The child DDS food groups were as follows: (1) grains, roots and tubers; (2) legumes and nuts; (3) dairy products; (4) flesh foods; (5) eggs; (6) vitamin A-rich fruits and vegetables; and (7) other fruits and vegetables. The child DDS has a range of 0 to 7. The rCSI is a simple tool applied in different contexts that assesses the frequency, in days within a 7-day period, and the severity of 5 coping strategies commonly used by households, when they cannot access enough food [25]. The 5 coping strategies are consuming less preferred foods, borrowing food, reducing meals, reducing portion sizes, and restricting adult’s food consumption to preserve children’s food consumption. As per recommendations, we weighted and summed the frequency responses to these strategies to create an index where higher scores indicate greater food insecurity. The rCSI range is 0 to 56. Rations of prepared food were distributed in this setting, so we asked how many days, within a 7-day period, the household relied on these wet rations. We asked how many meals were consumed in the household in the past 24-hour period.

### Household expenditure

We collected 30-day and 4-month household expenditure from a list of 10 food groups and 27 nonfood items. We then standardised all expenditure figures to a 30-day period and added them to calculate household total, food, and nonfood expenditure.

### Mother/caregiver characteristics and knowledge on health and nutrition

We asked mothers/caregivers their age in completed years, if they could read or not, if they had engaged in paid labour in the past year, and if they slept under a mosquito net in the past night. We assessed knowledge on health and nutrition topics using a list of 20 questions/statements covering 5 topics, for which respondents needed to answer yes or no to indicate whether they agreed with the statement (see S4 **Table**). Knowledge on health topics and nutrition was defined as the number of correctly answered questions.

### Vaccination coverage

We collected data on vaccination coverage, based on the Somalia Expanded Programme on Immunisation (EPI) schedule (see S2 **Table**), by checking the child’s health card at baseline, midline, and endline, or by mothers/caregivers recall if no health card was available or there was no entry for a particular antigen [26]. Bacillus Calmette–Guérin (BCG) vaccination status was assessed by the presence of a scar. Adverse events were not specifically recorded for trial purposes as there was no plausible reason to suspect that an increase in these would be associated with the intervention. During the trial, there was no indication of an increase in adverse vaccination events from routine health facility reports.

### Child age and anthropometry

We ascertained the children’s month and year of birth using a calendar of local events. We then randomly selected a day of the month to generate the date of birth. We estimated age in months as the difference between the day of data collection and the date of birth divided by 30.4.

We measured MUAC on the left arm to 1 mm precision using a standard MUAC insertion tape. We measured children’s MUAC in duplicate and entered both measures into the ODK questionnaire, which we designed to automatically estimate difference between these measurements and their mean. If the estimated difference was greater than 5 mm, we obtained a third measurement and estimated the mean using the pair of values with the lowest difference. We recorded presence of bilateral pitting oedema if an imprint remained in both feet after pressing them with the thumbs for 3 seconds. We defined acute malnutrition as the presence of a MUAC <12.5 cm and/or bilateral pitting oedema.

### Exclusive breastfeeding

We assessed exclusive breastfeeding in children aged <6 months using the WHO tool for IYCF practices [24] at R1, R3, and R9 visits. The WHO tool collects a 24-hour breastfeeding recall, as described by the child’s mother/caregiver, and information of liquids and food intake. We defined exclusive breastfeeding as reporting consumption of breastmilk in the past 24 hour while reporting no consumption of any other food or liquid.

### Child morbidity

We asked mother/caregivers whether the child had been unwell in the last 4 weeks or since the last round of data collection. We defined all-cause morbidity in children as having been reported unwell. Starting at the second round of data collection for children identified as having been unwell, we asked whether was due to diarrhoea or measles. We defined diarrhoea if the mother/caregiver reported the child had 3 or more loose or watery stools per day, and measles if the child had fever and maculopapular rash, with cough or coryza or conjunctivitis.

### Child mortality and verbal autopsy

For all the children, <5 years old, that died in the period between R1 and R9, we arranged for VA interviews, by trained staff, with their mothers or caregivers that reported having been with them in the period preceding death. VA interviews are designed to collect cause-of-death information—signs and symptoms—from deaths that occurred outside of health facilities and where death certification is weak or absent [27]. We adapted and shortened the 2016 WHO VA tool by removing nonrelevant questions, correcting skip patterns, and simplifying language to facilitate use by interviewers with no medical training. We calculated the probability of different causes of death using InterVA-5 software, which uses Bayesian reasoning to calculate the probability of cause of death categories based on the reported presence of signs, symptoms, and the circumstances of death [20]. For each death, InterVA-5 software reports the probability of up to 3 of the most likely causes. If the sum of these probabilities was less than 100, we allocated the remnant to an “indeterminate” category. We created a category called “lost to follow-up,” defined as the sum of the deaths lost to follow-up with VA interviews, each given a value of 100. We compiled and added all reported likelihoods for these causes of death to estimate the population level burden and estimated the population cause-specific mortality fractions (CSMFs) by dividing the population-level burden by the total number of deaths.

### Outcomes

The primary outcomes of the trial were assessed at the midline and endline time points. For the CCT intervention, these were measles vaccination coverage, defined as the proportion of children aged 9 to 59 months who had received MCV1, and “EPI vaccination coverage,” defined as the proportion of children aged <60 months who had received all their vaccinations according to the EPI schedule and their age (see Table 3). For the mHealth intervention, 2 additional primary outcomes were assessed: mother/caregiver’s knowledge of health and nutrition topics, defined as the mean number of correctly answered knowledge’s questions (see S1 Table), and dietary diversity of children aged 6 to 23 months, defined as the mean child DDS.

Following trial registration with ISRCTN, the name of the “EPI vaccination coverage” outcome was changed to “Timely Vaccination” to align better with terminology used in the literature, and “Pentavalent series completion,” defined as the proportion of children aged 12 to 23 months that had received all 3 does of Pentavalent vaccine, was added as an additional indicator of vaccination coverage.

The secondary outcomes of the trial were as follows: acute malnutrition incidence, assessed as a single failure; mortality incidence; all-cause morbidity incidence, assessed as a single failure; exclusive breastfeeding in children, defined as the proportion of exclusively breastfed children aged <6 months; and the CSMF obtained from VA interviews. Other outcomes of the trial included the following: all-cause morbidity, assessed as multiple failure; diarrhoea incidence, assessed as a single and multiple failure; measles incidence, assessed as a single failure; total, food, and nonfood household expenditure; and the food security indicators household diet diversity (HDDS) and the rCSI.

### Sample size

We estimated the sample size to observe differences in measles vaccination coverage and child DDS using the user-written *clustersampsi* function in Stata (StataCorp. 2015. Release 14. College Station, TX: StataCorp LP) [28]. As this function is designed for use with large numbers of clusters and because we did not include the Holm–Bonferroni adjustment for multiplicity, the calculated power was overestimated. We hypothesised that a greater measles vaccination coverage would be the results of the CCT intervention and that a greater child DDS would be the behavioural result of the mHealth intervention. Based on prior data [7], we expected a baseline measles vaccination coverage of around 64%. With 10 clusters per arm, with an average population of 65 children (aged <5 years) per cluster, and allowing for an alpha risk of 0.05 and an ICC of 0.06, we calculated we would have 80% power to detect a minimum increase of 18 percentage points in measles vaccination coverage. This minimum detectable difference laid below our hypothesised effect size of 20 percentage points. In addition, based on prior data [7], we expected a baseline child DDS of 3 with a standard deviation of 2. With 10 clusters per arm with an average population of 65 children (aged <5 years) per cluster, and allowing for an alpha risk of 0.05 and an ICC of 0.01, we calculated we would have 80% power to detect a minimum increase of 0.42 in child DDS.

### Data analysis

We undertook data analysis using Stata v17. Prevalence estimates at baseline were computed accounting for the trial cluster design using the Stata complex survey *svy* commands and differences in means and proportions were tested for using the *lincom* command. For variables collected at R1, R3, and R9 (baseline, midline, and endline), we assessed impact using mixed effects, multilevel, regression models: linear, logistic, or ordered logistic, depending on whether the outcome variables were continuous, categorical, or ordinal, respectively. Models included cluster and household variables as random-effects and dummy variables denoting the time point (baseline, midline, or endline) and intervention (CCT and mHealth) as fixed-effects in the model. All models included baseline data to account for any observed differences at baseline and were adjusted for age and sex. We conducted an available case analysis using mixed models, and the analysis is valid under the missing-at-random assumption. We used robust variance for calculation of standard errors. We tested for interactions between the 2 interventions and found no evidence of a significant effect for any outcome. We therefore report only the main effects in the results and provide a table of effects for the combined interventions in the annex. The absolute difference in risk for the vaccination coverage outcomes were calculated as the difference in regression adjusted contrasts, using the *margins* post-estimation command. The absolute difference in child diet diversity and maternal/caregiver knowledge scores was calculated using mixed effects linear regression. To provide a description of how the interventions effected the vaccination for each antigen, we also calculated the arithmetic difference-in-difference (DiD), by subtracting the baseline prevalence from the midline and endline values, and then subtracting the control prevalence from the intervention prevalence at each time point.

For assessing the impact on incidence, we estimated hazard ratios using Cox proportional hazards models, adjusting for child’s age and sex as covariates in the models, and using *svy* to calculate cluster-robust standard errors. For comparing probable causes of death, we used chi-squared tests, after we stratified the CSMF by arm. All analysis of intervention effects is “intention to treat,” meaning we compare clusters as randomised irrespective of whether participants received the intended intervention. *P* values were adjusted for multiple comparisons using the Holm–Bonferroni step-down procedure [29], but all confidence intervals are calculated at the 95% level. The adjustments were made based on all 72 tests that were conducted to test the impacts of the 2 interventions on the primary and secondary outcomes.

## Results

### Participants flow

**Fig 2:**
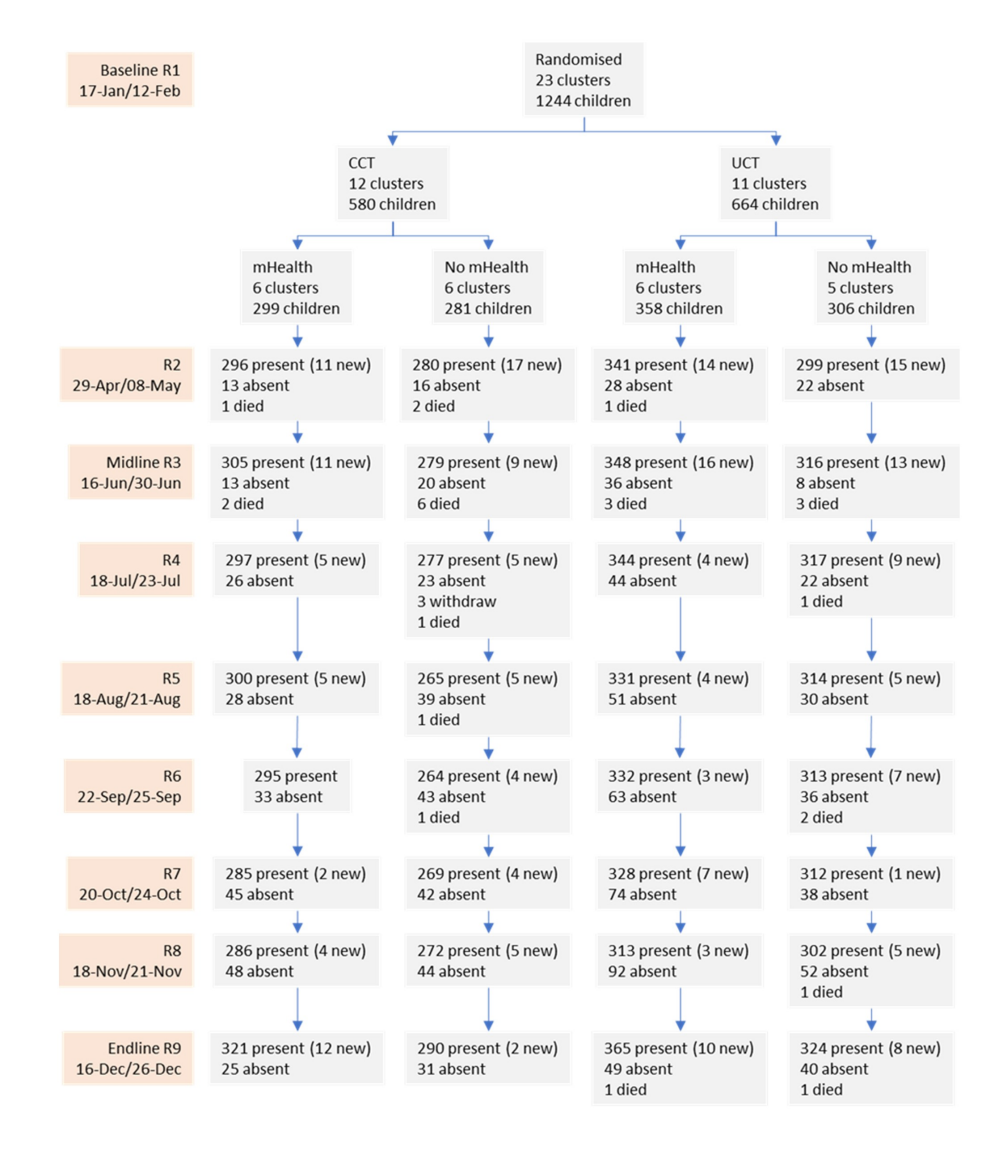
Study flowchart for children. CCT, conditional cash transfer; UCT, unconditional cash transfer.

**Figs 1** and **2** show the household and children flow for the 9 rounds of data collection. All households that were invited to participate enrolled in the study and all households receiving the CCT intervention met the conditionality and subsequently received the cash transfer. Overall, there was good household follow-up. Seven hundred and one households (90.6%) had data collected in all 9 rounds, and 745 households (96.3%) had data collected in at least 7 rounds. For children, a sample of 1,244 children were included in the study at baseline, and 225 children entered the study afterwards (57, 49, 23, 19, 14, 14, 17, and 32 for rounds 2, 3, 4, 5, 6, 7, 8, and 9, respectively). Between January 17 and December 26, we collected 11,545 observations.

Unfortunately, a total of 27 children died during that period, 17 boys and 10 girls (63% and 37%, respectively). The large proportion of these deaths (18 deaths, 66.7%) were reported in R2 and R3, which coincides with the Gu season (April to June) of long rains and flash floods in Somalia. The number of deaths in the CCT and control arms were comparable (14 and 13 deaths, respectively), but fewer deaths occurred in the mHealth arm than in the control arm (8 and 19 deaths, respectively).

### Baseline characteristics

**Tables 1 and 2** shows baseline characteristics of households, mothers/caregivers, and children. Most households reported being female headed and 15% were in a polygamous arrangement. The average household size was 5.1 members of which 1.6, 1.6, and 1.87 members were aged <5 years, 5 to 14 years, and 15 to 64 years, respectively. Households with elderly members, aged 65 and above, were less common with 1 elderly person for every 15 households. All households reported access to piped water, but a third paid for this access. A very small proportion of households reported access to handwashing facilities, while we observed soap ownership in only a third. Open defecation was rare as most households reported access to pit latrines. To collect water, households spent a mean of 43 minutes each day. On average, households reported consumption of 1.9 meals per day and receipt of wet food rations from a humanitarian organisation in 3 out of the past 7 days. Households reported, on average, a high dietary diversity with a consumption of about 7 out of 12 food groups in the past 24 hours. Households reported an average household expenditure of US$86.7 (95% CI [73.3; 100.0]) in the last 30 days, and food as their greatest expenditure category.

**Table 1:** Baseline characteristics of participant households randomised to receive the cash conditionality (CCT) or mHealth interventions.

	CCT (*n =* 371)		Control (*n* = 403)		mHealth (*n* = 404)		Control (*n* = 370)	
Household characteristics	mean / %	95% CI	mean / %	95% CI	mean / %	95% CI	mean / %	95% CI
Female head of household (%)	92.5	83.3; 96.8	93.8	83.5; 97.8	95.1	90.5; 97.5	91.1	77.7; 96.8
Polygamous arrangement (%)	15.6	12.6; 19.4	15.9	9.00; 26.5	13.6	9.54; 19.1	18.1	11.6; 27.2
Household size	5.22	4.89; 5.55	5.09	4.84; 5.35	5.18	4.90; 5.46	5.12	4.83; 5.42
Children aged <5 years	1.60	1.50; 1.71	1.66	1.57; 1.74	1.66	1.58; 1.75	1.60	1.50; 1.70
Children aged 5–14 years	1.61	1.44; 1.79	1.55	1.32; 1.79	1.63	1.41; 1.86	1.52	1.35; 1.70
Adults aged 15–64 years	1.92	1.77; 2.07	1.83	1.77; 1.90	1.82	1.70; 1.94	1.93	1.84; 2.02
Elderly aged 65+ years	0.09	0.05; 0.13	0.05	0.02; 0.08	0.06	0.03; 0.10	0.07	0.03; 0.11
Household total dependency ratio	1.95	1.81; 2.09	1.96	1.77; 2.15	2.07	1.93; 2.21	1.83	1.69; 1.96
Household child dependency ratio	1.90	1.75; 2.04	1.94	1.76; 2.12	2.03	1.89; 2.18	1.79	1.66; 1.92
Household aged dependency ratio	0.05	0.03; 0.08	0.02	0.01; 0.03	0.04	0.02; 0.06	0.03	0.01; 0.06
Time to collect water (minutes)	36.0	24.3; 47.7	50.3	29.0; 71.5	34.0	24.6; 43.4	53.7	31.7; 75.6
Household paid for water (%)	29.1	12.0; 55.2	26.3	14.9; 42.2	18.3	6.70; 41.2	37.8	22.7; 55.8
Handwashing facility in household (%)	1.08	0.21; 5.23	1.49	0.35; 6.17	2.23	0.68; 7.00	0.27	0.04; 1.87
Soap in household (%)	37.5	27.2; 49.1	33.8	21.4; 48.8	42.6	31.4; 54.6	27.8	18.7; 39.4
Open defecation (%)	2.70	0.61; 11.2	1.49	0.65l 3.37	1.73	0.90; 3.30	2.43	0.45; 12.0
Meals in last 24 hours	1.95	1.85; 2.05	1.94	1.83; 2.05	1.97	1.93; 2.01	1.91	1.77; 2.06
Consumption of wet rations (days in last 7 days)	2.16	1.51; 2.81	3.34	2.41; 4.28	2.80	1.95; 3.66	2.75	1.81; 3.69
Household DDS (12 food groups)	7.05	6.68; 7.41	7.22	6.76; 7.68	6.99	6.63; 7.36	7.29	6.83; 7.75
rCSI	20.4	18.1; 22.7	19.0	16.9; 21.1	18.2	16.2; 20.3	21.3	19.4; 23.1
Household expenditure in last 30 days (US$)	79.7	63.0; 96.5	93.1	74.1; 112.1	78.7	63.5; 94.0	95.4	74.4; 116.4
Household food expenditure in last 30 days (US$)	48.2	39.6; 56.9	60.4	46.8; 74.0	51.4	41.3; 61.5	58.0	43.5; 72.6
Household nonfood expenditure in last 30 days (US$)	31.5	22.0; 41.0	32.7	23.5; 42.0	27.3	18.6; 36.0	37.4	28.7; 46.1

CCT, conditional cash transfer; DDS, dietary diversity score; rCSI, reduced coping strategies index.

**Table 2:** Baseline characteristics of study participants randomised to receive the cash conditionality (CCT) or mHealth interventions^1^.

	CCT (*n =* 375)		Control (*n* = 405)		mHealth (*n* = 407)		Control (*n* = 373)	
Mother/Caregiver characteristics	mean / %	95% CI	mean / %	95% CI	mean / %	95% CI	mean / %	95% CI
Age (years)	31.7	30.5; 32.9	29.8	28.0; 31.6	31.0	29.5; 32.5	30.4	28.5; 32.3
Illiteracy (%)	88.5	80.6; 93.5	90.6	85.4; 94.1	89.2	83.3; 93.2	90.1	82.7; 94.5
Did paid work last year (%)	60.8	41.8; 77.0	56.3	41.0; 70.5	60.9	41.9; 77.1	55.8	40.5; 70.0
Slept under a mosquito net last night (%)	30.4	20.9; 42.0	21.2	15.8; 28.0	23.8	16.4; 33.3	27.6	19.5; 37.5
Knowledge score (maximum score of 20)	11.7	10.8; 12.7	11.9	10.6; 13.1	12.3	11.5; 13.1	11.3	9.9; 12.7
	**CCT (*n* = 580)**		**Control (*n =* 664)**		**mHealth (*n* = 657)**		**Control (*n* = 587)**	
**Children characteristics (aged 0–59 months)**	**mean / %**	**95% CI**	**mean / %**	**95% CI**	**mean / %**	**95% CI**	**mean / %**	**95% CI**
Age (months)	27.1	25.1; 29.1	27.8	26.5; 29.1	27.2	25.9; 28.5	27.8	25.8; 29.9
Male (%)	54.8	51.7; 57.9	51.1	47.6; 54.5	51.6	48.5; 54.7	54.2	50.3; 58.0
MUAC (cm)	14.2	14.0; 14.4	14.2	14.1; 14.4	14.3	14.1; 14.4	14.2	14.0; 14.4
Oedema (%)	0.58	0.20; 1.64	0.67	0.29; 1.54	0.67	0.28; 1.57	0.58	0.21; 1.60
Acute malnutrition (MUAC <12.5 cm and/or oedema)	5.98	4.43; 8.05	9.09	6.50; 12.58	8.14	6.31; 10.43	7.05	4.20; 11.58
Slept under a mosquito net last night (%)	28.5	17.6; 42.5	23.6	17.4; 31.3	22.7	15.8; 31.5	29.5	19.6; 41.7
Had health problems in last 4 weeks (%)	69.1	59.4; 77.5	62.7	51.9; 72.3	62.0	51.2; 71.7	69.9	60.5; 77.8
Received measles vaccination (%)	39.1	32.5; 46.2	45.2	40.0; 50.5	46.9	42.1; 51.7	37.3	31.2; 43.9
Received timely EPI vaccination (%)	17.8	10.7; 28.1	23.5	18.6; 29.2	23.0	16.6; 30.9	18.4	12.1; 27.0
Child dietary diversity score (7 food groups)	2.90	2.60; 3.19	2.82	2.53; 3.11	2.84	2.64; 3.05	2.87	2.49; 3.24

^1^As the trial used a 2 × 2 factorial design, participants who did not receive a particular intervention formed the control group for the main comparisons.

CCT, conditional cash transfer; EPI, Expanded Programme on Immunisation; MUAC, mid-upper arm circumference.

The mothers/caregivers from the sample of 774 households had an average age of 30 years (95% CI [29.5; 31.9]), 81% to 91% reported not been able to read, two-thirds reported to have done paid labour in the past year, and a quarter reported sleeping under a mosquito net the night before.

Children were on average 27 months old, and there was a 1.0:1.1 girl:boy sex ratio. One in 4 children were reported to sleep under a mosquito net the previous evening, and two-thirds were reported to have been ill in the previous 4-week period. Acute malnutrition affected 7.63% (95% CI [5.87, 9.85]) of the sample, 0.63% (95% CI [0.33, 1.21]) presented oedema, and they had a mean MUAC of 14.2 cm (95% CI [14.1, 14.4]). Measles vaccination was low at 42.4% (95% CI [38.0, 46.9]), and only 20.8% (95% CI [16.1, 26.4]) had received all vaccinations according to the age schedule. On average, children consumed food from 2.85 food groups (95% CI [2.64, 3.06]), and those aged 6 to 23 months consumed food from 3.05 food groups (95% CI [2.79, 3.31]).

### Intervention exposure

All households in the study were registered to benefit from cash transfers and received their cash transfers in a timely manner. **Table 3** shows that the participant’s exposure to the CCT and mHealth interventions was high throughout the study. We detected minor leakage of the intervention between the study arms, with 6% of households in the control arm reporting having heard a mHealth audio message between baseline and round 2. However, this reported leakage fell markedly in subsequent data collection rounds.

**Table 3:** Participants reported exposure to interventions.

		Round							
		2	3 (midline)	4	5	6	7	8	9 (endline)
Child had study health card (%)	CCT^1^	94.5	95.1	93.3	88.5	96.1	95.5	95.5	99.0
	Control	0.45	0.00	0.14	0.42	0.00	0.28	0.41	0.00
Mother/caregiver listened to at least 1 mHealth message (%)	mHealth	95.1	99.5	95.2	98.6	99.4	98.5	97.0	98.9
	Control	5.76	2.52	0.86	0.30	0.31	0.00	0.00	2.03

^1^CCT, conditional cash transfer.

### Primary outcomes

**Table 4** show the impact on the 5 primary outcomes of the study: measles vaccination, pentavalent series completion, timely EPI vaccination, maternal/caregiver knowledge, and child dietary diversity. The CCT intervention significantly improved measles vaccination coverage from 39.2% to 77.5% and Pentavalent series completion from 44.2% to 77.5%. This improvement was observed at midline and had increased further by endline. Comparing prevalence changes between the study arms using a DiD approach, we found an increase in crude measles coverage of 39.1% (95% CI [24.5, 53.8]) by endline (see S5 **Table**). S6 **Table** shows the crude vaccination coverage for all antigens in children aged 0 to 59 months at baseline, midline, and endline for the CCT and control arms. At endline, compared with the control group, the group receiving the CCT intervention showed significantly greater coverage in all vaccinations, except oral polio vaccine 0 (OPV 0) and BCG. However, we found that CCT did not improve timely EPI vaccination coverage.

There was no evidence that the mHealth intervention improved mother’s knowledge, any of the vaccination outcomes, or child diet diversity (see Table 4 for hypothesis testing and S7 Table for DiD prevalence values).

**Table 4:** Effect of the cash transfer conditionality (CCT) and mHealth interventions on primary outcomes.

	*Midline (June 2019*, *after 3 months of intervention)*						*Endline (December 2019*, *after 9 months of intervention)*						ICC^6^
Intervention outcomes	Odds ratio	95% CI	*P*	Absolute difference (% points or score)	95% CI	Intervention Interaction *p*	Odds ratio	95% CI	*p*	Absolute difference (% points or score)	95% CI	Intervention Interaction *p*	
**CCT vs Control**													
Measles vaccination^1^	11.70*	5.25; 26.08	<0.001	33.2	22.3; 44.1	0.250	28.15*	13.90; 57.01	<0.001	39.0	27.2; 50.8	0.850	0.438
Pentavalent series completion^2^	8.85*	2.63; 29.79	<0.001	28.0	13.0; 43.1	0.370	33.77*	11.03; 103.36	<0.001	42.9	28.8; 57.0	0.317	0.583
Timely EPI vaccination^3^	1.23	0.39; 3.86	0.720	2.3	−11.2; 15.6	0.575	0.50	0.18; 1.38	0.183	−6.0	−17.0; 5.0	0.371	0.458
**mHealth vs Control**													
Measles vaccination^1^	0.45	0.21; 0.92	0.029	−13.4	−24.1; −2.66	0.250	0.39	0.15; 0.97	0.043	−15.0	−26.8; −3.1	0.850	0.438
Pentavalent series completion^2^	0.78	0.27; 2.34	0.639	−3.6	−0.18; 0.10	0.370	0.93	0.33; 2.57	0.883	−1.0	−0.15; 0.13	0.317	0.583
Timely EPI vaccination^3^	0.65	0.20; 2.11	0.478	−5.0	−18.7; 8.8	0.575	0.46	0.18; 1.18	0.108	−8.7	−19.2; 1.8	0.371	0.458
Maternal/caregiver knowledge^4^	1.06	0.17; 6.61	0.947	−0.10	−2.10; 1.90	0.428	1.32	0.25; 7.11	0.746	0.21	−1.59; 2.01	0.776	0.197
Child dietary diversity^5^	1.95	0.87; 4.37	0.107	0.54	−0.09; 1.18	0.706	2.13	1.00; 4.55	0.050	0.56	−0.02; 1.15	0.097	0.137

In all analyses except maternal knowledge, the odds ratios are based on including both interventions in the model and adjusting for child age and sex, and the baseline value of the outcome variable. For maternal knowledge, the odds ratio was based on including both interventions in the model and adjusting for maternal age. Unadjusted percentages, scores, and n are provided in S5 and S7 Tables.

* Significant after Holm–Bonferroni correction.

^1^ We assessed impact in children aged 9 to 59 months using logistic regression.

^2^ Impact was assessed in children aged 12 to 23 months using logistic regression.

^3^ We assessed impact in children aged 0 to 59 months using logistic regression. EPI, Expanded Programme on Immunization.

^4^ We assessed impact in mothers/caregivers using ordered logistic regression.

^5^ We assessed impact in children aged 6 to 23 months using ordered logistic regression.

^6^ ICC, intracluster correlation coefficient; EPI, Expanded Programme on Immunization.

### Secondary outcomes and other outcomes

**Table 5** shows the interventions association with household expenditure, food security, and exclusive breastfeeding. The CCT intervention did not affect household food and nonfood expenditure. The CCT intervention appeared to increase household dietary diversity at endline, but this did not remain significant following the Holm–Bonferroni correction. The CCT intervention was not associated with household reliance on coping strategies.

**Table 5:** Intervention effects on household expenditure, food security, and exclusive breastfeeding.

	CCT vs Control^4^						mHealth vs Control					
	Midline			Endline			Midline			Endline		
Categories	coef.	95% CI	*P*	coef.	95% CI	*P*	coef.	95% CI	*P*	coef.	95% CI	*P*
Household expenditure												
Food (US$)^1^	−3.75	−27.48; 19.98	0.757	0.14	−20.19; 20.46	0.989	11.01	−13.52; 35.53	0.379	13.18	−7.77; 34.12	0.218
Nonfood (US$)^1^	−3.03	−19.72; 13.66	0.722	−1.83	−16.79; 13.12	0.810	8.21	−8.42; 24.86	0.333	6.49	−8.35; 21.33	0.391
Food security indicators												
HDDS^2^	1.75	−0.68; 4.51	0.247	1.95	1.04; 3.65	0.038	2.47	0.99; 6.14	0.038	3.75*	2.04; 6.88	<0.001
rCSI^2^	0.92	0.24; 3.49	0.901	0.94	0.27; 3.28	0.917	1.33	0.37; 4.77	0.664	1.96	0.55; 6.95	0.297
Infant feeding												
Exclusive breastfeeding^3^	1.04	0.14; 7.62	0.969	0.69	0.11; 4.17	0.685	1.17	0.23; 5.89	0.850	2.12	0.33; 13.71	0.432

In all analyses, child age and sex were included as covariates, and the OR was adjusted for baseline values of the outcome variable. Midline data collection took place in June 2019, after 3 months of intervention, and endline data collection took place in December 2019, after 9 months of intervention.

* Significant after Holm–Bonferroni correction.

^1^ We assessed impact using linear regression and report regression coefficients. Coefficients >1 indicate a positive impact of the intervention on expenditure.

^2^ We assessed impact using ordered logistic regression and report odds ratios. HDDS, household diet diversity score; rCSI, reduced coping strategy index.

^3^ Impact was assessed using logistic regression, and we report adjusted odds ratios.

^4^CCT, conditional cash transfer; coef., coefficient.

The mHealth intervention was not associated with household expenditure but significantly increased household DDS by endline from a mean of 7.0 to 9.5. There was no effect on household coping strategies or exclusive breastfeeding in children aged <6 months.

**Table 6** presents the survival analysis for mortality, morbidity, and acute malnutrition. The CCT intervention reduced the risk of measles infection, consistent with the increase in vaccination coverage described above. However, these effects were no longer significant after the Holm–Bonferroni correction. The CCT intervention did not affect the risk of all-cause morbidity or the risk of diarrhoea, when assessed as either a single failure or multiple failures, and had no impact on the risk of dying.

**Table 6:** Intervention effects on the risk of mortality and morbidity.

	CCT vs Control^1^			mHealth vs Control		
	Hazard Ratio	95% CI	*p*	Hazard Ratio	95% CI	*p*
Mortality	1.22	0.43; 3.47	0.704	0.38	0.15; 0.96	0.040
Acute malnutrition	1.73	0.98; 3.07	0.060	0.80	0.42; 1.54	0.492
All-cause morbidity (single failure)	0.89	0.54; 1.47	0.640	1.08	0.66; 1.76	0.747
All-cause morbidity (multiple failure)	0.95	0.55; 1.63	0.844	0.86	0.52; 1.42	0.530
Diarrhoea (single failure)	0.82	0.40; 1.69	0.579	0.58	0.29; 1.13	0.103
Diarrhoea (multiple failure)	1.01	0.44; 2.29	0.986	0.57	0.27; 1.21	0.137
Measles (single failure)	0.32	0.10; 0.99	0.048	1.81	0.67; 4.85	0.225

We undertook all analyses using Cox proportional hazards models.

We included children’s age and sex as covariates in all models and adjusted the standard errors for the study’s clustered design.

None remain significant after Holm–Bonferroni correction.

^1^CCT, conditional cash transfer.

The mHealth intervention did not affect the risks of acute malnutrition, measles, all-cause morbidity, and diarrhoea, but there was a suggestion that it may have reduced the risk of death. There were a total of 27 deaths recorded in study children with 8 occurring in the mHealth intervention arm and 19 in the control, with an overall death rate of 0.2 deaths/100 child-months. However, after the Holm–Bonferroni correction was applied, this difference was not significant. Out of the 27 recorded deaths, we gathered VA data from 24 children (15 male and 9 female), an overall follow-up of 88.9%. The mothers/caregivers of the remaining 3 children could not be traced. The 3 leading probable causes of death were diarrhoeal diseases, measles, and severe malnutrition, accounting for 55% of the total CSMF. Causes of death are ranked in S3 **Table**. We stratified the CSMF by sex and interventions, but due to the small number of deaths that occurred in the study, we did not test for differences between groups.

## Discussion

This study was among the first trials in Africa to demonstrate a beneficial impact of a CCT intervention on child immunisation. Introduction of a single time point conditionality and provision of an enhanced home-based health record card had a highly significant positive impact on vaccination with improvements in both measles (MCV1) vaccination coverage and pentavalent series completion. The mHealth intervention was associated with an increase in household diet diversity, despite no evidence that knowledge had improved. To the best of our knowledge, this is the first study to demonstrate that mHealth interventions may improve household diet diversity when combined with a multipurpose UCT programme. However, no change in child diet diversity or any other primary outcome was observed, and there was no improvement in exclusive breastfeeding.

The strong impact of cash transfer conditionality on uptake of vaccination services is consistent with other studies that have provided evidence that conditional transfers can enhance the use of health services and improve health outcomes [8,12]. However, our findings contrast with a recent review of CCT in Africa, which showed no impact on immunisation coverage [30]. Evidence on the impact of home-based health records on vaccination is very limited and inconclusive, although their use is recommended by WHO, especially in remote and hard-to-reach populations [21]. The design of this study did not permit us to determine the relative contribution of record cards and conditionality to the intervention effect.

The selection and calculation of vaccination indictors for use in different studies is somewhat variable [31]. Here, we chose to use “measles vaccination coverage” in children above 9 months due to the importance of measles infections for child mortality in this and other emergency and displacement contexts, its use as an indicator of programme adequacy by the Sphere Project, and the finding from a previous trial in the same area that coverage was low [7,32,33]. We also used “Pentavalent series completion” to indicate the overall functioning of routine EPI vaccination and allow comparison with the global DTP3 coverage indicator used by WHO and the DPT series completion used as a Sphere indicator [32,34]. Lastly, we measured “Timely vaccination” to provide information of the ability of the EPI system to provide age-appropriate vaccination.

Timely vaccination (also referred to as on-time vaccination or its reverse, delayed vaccination) is important to assess as delayed vaccination may lead to an increase in mortality and it increases the fraction of a population that requires vaccination in order to eliminate a disease [35,36]. When measured in other contexts, the proportion of children receiving timely vaccination has also often been low [37,38]. Analysis by antigen and child age revealed that administration of OPV0 and BCG were low in the CCT intervention group, and this accounted for the lack of improvement in the Timely Vaccination indicator despite the large improvements in age-appropriate coverage of other antigens. A lack of improvement in OPV0 coverage has also been reported in a CCT trial in Nigeria [39]. OPV0 was likely to be missed out as infants were frequently offered BCG, OPV1, and Pentavalent 1 on their first to a health facility, rather than BCG and OPV0. This suggests that further work on staff training and support is required, as well as a review of the immunization schedule and community awareness and acceptability.

Achieving full vaccination is challenging in this context for a range of reasons, including weak health infrastructure, financial and human resource constraints, insecurity, a highly mobile population, as well as knowledge of and attitudes towards vaccination among caretakers. Evidence-based approaches to identify and remove barriers to vaccination are recommended as part of the Immunization 2030 global strategy [40]. Since the end of this study, the Government of Somalia has updated its EPI policy, which continues to prioritise routine immunisation for children under 1 year while also promoting vaccination for other eligible groups, including children 12 to 24 months who were previously unvaccinated. We observed no impact on child morbidity during the follow-up period of the study, which is in line with a recent review that showed no consistent effect of CCT on child health outcomes in Africa [30]. However, the beneficial impacts of enhanced immunisation would be likely to manifest over the longer term and be of particular benefit during the periodic outbreaks of measles and other diseases that have afflicted this population [33].

mHealth interventions have been demonstrated to have potential to improve multiple health indicators in low-income countries [41]. We examined a range of outcomes here but achieved mixed results. Knowledge score did not improve, although in both control and intervention arms, it increased by 2.5 points between baseline and midline, suggesting a possible learning effect induced by the asking of the questions at baseline. No increase was seen between midline and endline. Little evidence exists on the effectiveness of audio message approaches, as used in this study. Evidence on the impact of mHealth on diet diversity in other contexts is sparse, although some alternative approaches have been shown to increase vegetable consumption [42]. Given the mixed results reported here, further work to refine and test the use of mHealth audio messages in this and similar contexts is warranted with further exploration of the links between knowledge and practice.

The partnership between an NGO that had been operational in the area for many years and an academic institution with a strong track record in research in fragile contexts provided the necessary expertise to conduct this trial in challenging circumstances. Regular visits by the researchers to the field sites and the strong working relationships between the academic study coordinator and the NGO field coordinator were essential to the robust running of the trial. Periodic, monthly follow-up visits allowed close monitoring of participants with regular data collection and for the calculation of incidence as well as prevalence indicators.

Use of both health record cards and caregiver recall to determine child vaccination status allowed for measurements to be made in the whole population sample. Only a small proportion possessed record cards at baseline, so if we had relied on card records only, it is likely to have resulted in selection of a biased sample. However, as record cards were issued as part of the CCT intervention, there was an unavoidable difference between the CCT and control arms in how vaccination data were collected after the baseline. It is therefore possible that the greater use of record cards in the CCT arm may have resulted in improved reporting of vaccinations and a resulting measurements bias. However, a recent study has demonstrated that while results from studies are inconsistent, parental recall compared to record-based data in East Africa may be reliable [43]. In addition, the large increases in vaccination coverage observed in the CCT arm between midline and endline (when record cards were used at both time points) argue that the observed intervention effect was not due to improved reporting during data collection.

Working with national companies experienced in production and broadcasting of mHealth messages helped to ensure the quality of the productions and allowed for messages to be recorded in both of the commonly used local languages. The process of mHealth message design was originally conceived as involving successive iterative cycles of design and message testing. Due to limitations in the available time, the intended testing process had to be curtailed, and, while messages were reviewed by the local study team, it was not possible to pretest all messages with participant volunteers before broadcast. Additionally, the BCC approach was ambitious, aiming to address multiple behaviours simultaneously, instead of focusing on few simple messages at a time. However, engagement with the mHealth messages remained high throughout the study, suggesting the general desirability of the messages among participants.

The use of a 2 × 2 factorial design allowed the testing of 2 important interventions and was therefore cost-efficient. However, the study design also required a large number of comparisons and significance tests. The use of Holm–Bonferroni correction is recommended in situations where a large number of tests are required, and we adopted it for the analysis of this trial to reduce the risk of type 1 errors [29].

Our data show that including a simple, one time point conditionality in a humanitarian cash transfer programme can increase the uptake of proven life-saving interventions, such as child vaccination. Further work to understand the contribution of home-based health record cards and how CHW interacted with them would be useful and contribute to optimising their design. Use of mHealth messages may be useful for increasing expenditure on food and diet diversity during cash transfer programmes. However, in this context, they failed to achieve any direct health benefits, despite achieving high engagement. Future studies should include iterative message design and intensive pretesting of content and format with participant volunteers. This would help to maximise the chances of utilising this potentially desirable modality to achieve health and nutrition impacts in this complex context.

## Supporting information

S1 CONSORT Checklist CONSORT 2010 checklist of information study checklist prepared using CONSORT guidelines. (DOC)

S1 Table Data collected during each of the 9 rounds (R1-R9). (DOCX)

S2 Table Expanded programme of immunisation schedule in Somalia. (DOCX)

S3 Table Cause-specific mortality fraction within each trial arm ranked by proportional contribution. (DOCX)

S4 Table List of 20 statements used to assess mother/caregiver knowledge. (DOCX)

S5 Table Unadjusted primary outcome indicators at baseline, midline, and endline in the conditional cash transfer vs control comparison. (DOCX)

S6 Table Crude vaccination coverage of children aged 0–59 months at baseline, midline, and endline in the conditional cash transfer vs control comparison. (DOCX)

S7 Table Unadjusted primary outcome indicators at baseline, midline, and endline in the mHealth vs control comparison. (DOCX)

S8 Table Combined effects of the conditional cash transfer (CCT) and mHealth interventions on primary outcomes. (DOCX)

S1 Study Protocol CINS study protocol. (DOC)

## Abbreviations

BCC: behaviour change communication
BCG: Bacillus Calmette–Guérin
CBI: cash-based intervention
CCT: conditional cash transfer
CHW: community health worker
CINS: Cash for Improved Nutrition in Somalia
CSMF: cause-specific mortality fraction
DDS: dietary diversity score
DiD: difference-in-difference
EPI: Expanded Programme on Immunisation
IDP: internally displaced people
IVR: interactive voice response
IYCF: infant and young child feeding
MEB: minimum expenditure basket
mHealth: mobile health
MUAC: mid-upper arm circumference
ODK: Open Data Kit
rCSI: reduced coping strategies index
REFANI: Research on Food Assistance for Nutritional Impact
UCL: University College London
UCT: unconditional cash transfer
VA: verbal autopsy
WASH: water, sanitation, and hygiene
WHO: World Health Organisation
