# Supplementary material for: Evaluation of conditional cash transfers and mHealth audio messaging in reduction of risk factors for childhood malnutrition in internally displaced persons camps in Somalia: A 2 × 2 factorial cluster-randomised controlled trial
Source: PLoS Med. 2023 Feb 27;20(2):e1004180. doi: 10.1371/journal.pmed.1004180 (PMC9970051; doi:10.1371/journal.pmed.1004180)
Supplement: S1 Table — (DOCX) [file pmed.1004180.s002.docx]

**Table A1.** Data collected during each of the nine rounds (R1-R9) .

|  | **Baseline**  **Jan-Feb 2019** |  | **Midline**  **Jun 2019** |  |  |  |  |  | **Endline**  **Dec 2019** |
| --- | --- | --- | --- | --- | --- | --- | --- | --- | --- |
|  | **R1** | **R2** | **R3** | **R4** | **R5** | **R6** | **R7** | **R8** | **R9** |
| **Household** |  |  |  |  |  |  |  |  |  |
| Demographics | X |  |  |  |  |  |  |  |  |
| Assets | X |  |  |  |  |  |  |  |  |
| Expenditure | X |  | X |  |  |  |  |  | X |
| WASH | X |  | X |  |  |  |  |  | X |
| Food security | X |  | X |  |  |  |  |  | X |
| M&E cash transfers | X | X | X | X | X | X | X | X | X |
| M&E mHealth | X | X | X | X | X | X | X | X | X |
| **Mothers/caregivers** |  |  |  |  |  |  |  |  |  |
| Age | X |  |  |  |  |  |  |  |  |
| Literacy | X |  |  |  |  |  |  |  |  |
| Work | X |  |  |  |  |  |  |  |  |
| Knowledge of health topics | X |  | X |  |  |  |  |  | X |
| **Children aged <5 years** |  |  |  |  |  |  |  |  |  |
| Vaccination coverage | X |  | X |  |  |  |  |  | X |
| Breastfeeding | X |  | X |  |  |  |  |  | X |
| Anthropometry/Malnutrition | X | X | X | X | X | X | X | X | X |
| Illness | X | X | X | X | X | X | X | X | X |
| Mortality | X | X | X | X | X | X | X | X | X |
| Possession of Health card | X | X | X | X | X | X | X | X | X |
|  |  |  |  |  |  |  |  |  |  |
