# Supplementary material for: Evaluation of conditional cash transfers and mHealth audio messaging in reduction of risk factors for childhood malnutrition in internally displaced persons camps in Somalia: A 2 × 2 factorial cluster-randomised controlled trial
Source: PLoS Med. 2023 Feb 27;20(2):e1004180. doi: 10.1371/journal.pmed.1004180 (PMC9970051; doi:10.1371/journal.pmed.1004180)
Supplement: S2 Table — (DOCX) [file pmed.1004180.s003.docx]

**Table A2.** Expanded Programme of Immunisation Schedule in Somalia.

| **Recommended age** | **Vaccines** |
| --- | --- |
| At Birth | - Bacillus Calmette–Guérin vaccine - Oral polio vaccine 0 |
| 6 weeks (42 days) | - Pentavalent vaccine 1 - Oral polio vaccine 1 |
| 10 weeks | - Pentavalent vaccine 2 - Oral polio vaccine 2 |
| 14 weeks | - Pentavalent vaccine 3 - Oral polio vaccine 3 - Inactivated polio vaccine |
| 9 months | - Measles vaccine |
| Pentavalent vaccine provides immunisation against Diphtheria, Pertussis, Tetanus, Hepatitis B and Haemophilus Influenza. | |
