# Supplementary material for: Evaluation of conditional cash transfers and mHealth audio messaging in reduction of risk factors for childhood malnutrition in internally displaced persons camps in Somalia: A 2 × 2 factorial cluster-randomised controlled trial
Source: PLoS Med. 2023 Feb 27;20(2):e1004180. doi: 10.1371/journal.pmed.1004180 (PMC9970051; doi:10.1371/journal.pmed.1004180)
Supplement: S3 Table — (DOCX) [file pmed.1004180.s004.docx]

**Table A3.** Cause specific mortality fraction within each trial arm ranked by proportional contribution.^1^

|  |  | **CCT vs Control^2^** | | **mHealth vs Control** | | **Sex** | |
| --- | --- | --- | --- | --- | --- | --- | --- |
| **Probable cause of death** | **Total** | **CCT** | **Control** | **mHealth** | **Control** | **Boys** | **Girls** |
| Diarrhoeal diseases | 36.2 | 48.9 | 22.6 | 41.5 | 34.0 | 28.6 | 49.2 |
| Measles | 11.0 | 14.1 | 7.6 | 0.0 | 15.6 | 5.8 | 19.8 |
| Severe malnutrition | 7.7 | 3.1 | 12.7 | 0.0 | 11.0 | 7.9 | 7.5 |
| Sickle cell with crisis | 7.0 | 0.0 | 14.5 | 12.9 | 4.7 | 5.8 | 9.0 |
| Indeterminate | 6.5 | 4.4 | 8.8 | 1.9 | 8.4 | 7.7 | 4.5 |
| Malaria | 5.8 | 4.1 | 7.6 | 19.5 | 0.0 | 9.2 | 0.0 |
| Acute respiratory infection (incl. pneumonia) | 3.7 | 7.1 | 0.0 | 0.0 | 5.2 | 5.8 | 0.0 |
| Stroke | 3.6 | 0.0 | 7.5 | 12.3 | 0.0 | 5.8 | 0.0 |
| Pertussis | 3.3 | 6.4 | 0.0 | 0.0 | 4.7 | 5.2 | 0.0 |
| Acute cardiac disease | 2.5 | 4.9 | 0.0 | 0.0 | 3.6 | 4.0 | 0.0 |
| Sepsis (non-obstetric) | 1.6 | 0.0 | 3.2 | 0.00 | 2.2 | 2.5 | 0.0 |
| Lost-to-follow-up | 11.1 | 7.1 | 15.9 | 12.5 | 10.5 | 11.8 | 10.0 |
| **Total** | **100.0** | **100.0** | **100.0** | **100.0** | **100.0** | **100.0** | **100.0** |

^1^ Cause specific mortality fractions are given as percentages

^2^ CCT, conditional cash transfer
