# Supplementary material for: Evaluation of conditional cash transfers and mHealth audio messaging in reduction of risk factors for childhood malnutrition in internally displaced persons camps in Somalia: A 2 × 2 factorial cluster-randomised controlled trial
Source: PLoS Med. 2023 Feb 27;20(2):e1004180. doi: 10.1371/journal.pmed.1004180 (PMC9970051; doi:10.1371/journal.pmed.1004180)
Supplement: S4 Table — (DOCX) [file pmed.1004180.s005.docx]

**Table A4.** List of 20 statements used to assess mother/caregiver knowledge.

| **Topic** | **Statement** |
| --- | --- |
| IYCF | The first milk or colostrum from the mother is not good for babies and can cause sickness |
|  | Breastfeeding immediately after birth provides a child with adequate nutrition |
|  | Feeding children with a range of different foods every day is a good way to provide them with enough nutrients to grow well |
|  | Feeding a new-born with infant formula is as safe and nutritious as breastfeeding them |
| Sickness | A child that is unable to drink or breastfeed should be quickly taken to the health clinic |
|  | You should reduce the intake of liquids and food when a child has diarrhoea |
|  | Reading the Quran is the only action you need to take if a child gets very sick |
|  | Smelling ‘uris’ is the main cause of diarrhoea in children |
| Vaccination | Vaccination can prevent a child catching polio, whooping cough, and mumps |
|  | A child can be safely vaccinated against several diseases at the same time |
|  | Vaccinating children can cause them to grow more slowly |
|  | Drinking goat's milk can prevent a child getting measles |
| WASH | Using latrines helps to avoid spreading germs to other people |
|  | Chlorine is sometimes added to water to make it safe to drink |
|  | You should only use soap to wash your hands after using the latrine |
|  | You should wash your hands with soap before preparing any meal |
| Malnutrition | Children should only be taken to a clinic to be treated for malnutrition when they are thin enough to see their bones |
|  | Children can develop malnutrition because of an illness |
|  | A child that looks swollen or has loose skin, is likely to have acute malnutrition |
|  | Children can develop malnutrition if they don’t get enough different types of food |
|  | |
