# Supplementary material for: Evaluation of conditional cash transfers and mHealth audio messaging in reduction of risk factors for childhood malnutrition in internally displaced persons camps in Somalia: A 2 × 2 factorial cluster-randomised controlled trial
Source: PLoS Med. 2023 Feb 27;20(2):e1004180. doi: 10.1371/journal.pmed.1004180 (PMC9970051; doi:10.1371/journal.pmed.1004180)
Supplement: S5 Table — (DOCX) [file pmed.1004180.s006.docx]

**Table A5.** Unadjusted primary outcome indicators at baseline, midline, and endline, in the conditional cash transfer vs control comparison.

|  | Baseline (Jan-Feb 2019) | | | | Midline (Jun 2019) | | | | Endline (Dec 2019) | | | |
| --- | --- | --- | --- | --- | --- | --- | --- | --- | --- | --- | --- | --- |
|  | Control | | CCT | | Control | | CCT | | Control | | CCT | |
|  | n | % or  mean | n | % or  mean | n | % or  mean | n | % or  mean | n | % or  mean | n | % or  mean |
| Measles  vaccination^1^ (%) | 546 | 53.5 | 480 | 47.1 | 545 | 61.1 | 494 | 87.3 | 505 | 60.79 | 478 | 93.5 |
| Pentavalent series vaccination^2^ (%) | 149 | 59.7 | 156 | 44.2 | 161 | 64.0 | 142 | 77.5 | 145 | 55.9 | 121 | 86.8 |
| Timely EPI  Vaccination^3^ (%) | 664 | 24.3 | 580 | 17.9 | 673 | 23.2 | 587 | 19.9 | 689 | 22.5 | 614 | 10.6 |

^1^ We assessed this outcome in children aged 9-59 months.

^2^ We assessed this outcome in children aged 12-23 months.

^3^ We assessed this outcome in children aged 0-59 months. EPI, Expanded Programme on Immunization
