# Supplementary material for: Evaluation of conditional cash transfers and mHealth audio messaging in reduction of risk factors for childhood malnutrition in internally displaced persons camps in Somalia: A 2 × 2 factorial cluster-randomised controlled trial
Source: PLoS Med. 2023 Feb 27;20(2):e1004180. doi: 10.1371/journal.pmed.1004180 (PMC9970051; doi:10.1371/journal.pmed.1004180)
Supplement: S6 Table — (DOCX) [file pmed.1004180.s007.docx]

**Table A6.** Crude vaccination coverage of children aged 0-59 months at baseline, midline, and endline, in the conditional cash transfer vs control comparison.

|  | **Baseline (R1, Jan-Feb 2019)** | | | | **Midline (R3, Jun 2019)** | | | | **Endline (R9, Dec 2019)** | | | | **DiD** | | | **DiD** | | |
| --- | --- | --- | --- | --- | --- | --- | --- | --- | --- | --- | --- | --- | --- | --- | --- | --- | --- | --- |
|  | **Control** | | **CCT** | | **Control** | | **CCT** | | **Control** | | **CCT** | | **(R3 – R1)** | | | **(R9 – R1)** | | |
|  | % | 95% CI | % | 95% CI | % | 95% CI | % | 95% CI | % | 95% CI | % | 95% CI | diff | 95% CI | *p* | diff | 95% CI | *p* |
| BCG scar | 40.6 | 33.4; 48.2 | 32.1 | 24.1; 41.4 | 43.3 | 33.5; 53.6 | 34.3 | 27.8; 41.5 | 40.0 | 35.5; 44.7 | 30.4 | 22.0; 40.4 | -0.46 | -16.5; 15.6 | 0.953 | -1.12 | -14.6; 12.3 | 0.865 |
| OPV 0 | 50.6 | 39.9; 61.3 | 35.2 | 24.2; 48.1 | 47.7 | 30.9; 65.0 | 59.8 | 43.6; 74.2 | 56.8 | 44.9; 68.0 | 44.0 | 38.0; 50.1 | 27.5 | 0.16; 54.9 | 0.049 | 2.52 | -17.8; 22.8 | 0.799 |
| Penta 1/OPV 1 | 63.2 | 59.4; 66.8 | 51.3 | 41.2; 61.3 | 67.2 | 63.0; 71.1 | 74.7 | 69.2; 79.5 | 70.9 | 61.9; 78.6 | 78.8 | 73.1; 83.5 | 19.4 | 8.65; 30.1 | 0.001 | 19.7* | 9.30; 30.1 | <0.001 |
| Penta 2/OPV 2 | 55.9 | 51.9; 59.9 | 44.4 | 37.3; 51.7 | 62.2 | 56.9; 67.2 | 70.3 | 64.3; 75.6 | 60.2 | 49.9; 69.6 | 75.3 | 69.3; 80.4 | 19.6* | 11.0; 28.2 | <0.001 | 26.6* | 13.6; 39.7 | <0.001 |
| Penta 3/OPV 3 | 51.2 | 45.8; 56.6 | 39.6 | 32.6; 46.9 | 58.8 | 51.7; 65.5 | 66.7 | 60.5; 72.4 | 52.6 | 44.7; 60.4 | 71.8 | 65.6; 77.3 | 19.6* | 10.0; 29.3 | <0.001 | 30.9* | 18.7; 43.0 | <0.001 |
| IPV | 48.6 | 43.5; 53.8 | 38.5 | 31.6; 46.0 | 56.4 | 48.3; 64.2 | 60.7 | 51.8; 68.9 | 49.1 | 41.3; 56.9 | 65.4 | 57.6; 72.4 | 14.4 | 1.60; 27.2 | 0.029 | 26.4* | 13.1; 39.7 | <0.001 |
| Measles | 45.2 | 40.0; 50.4 | 39.2 | 32.6; 46.3 | 53.0 | 46.0; 59.8 | 77.5 | 73.8; 80.9 | 52.9 | 44.8; 60.9 | 82.2 | 78.7; 85.3 | 30.5* | 19.8; 41.2 | <0.001 | 35.2* | 23.0; 47.5 | <0.001 |

BCG: Bacillus Calmette-Guérin, Penta: Pentavalent vaccine, OPV: oral cholera vaccine, IPV: inactivated polio vaccine

* Significant after Holm-Bonferroni correction.

DiD: Difference-in-difference; obtained as the simple arithmetic difference between rounds and intervention arms.
