# Supplementary material for: Evaluation of conditional cash transfers and mHealth audio messaging in reduction of risk factors for childhood malnutrition in internally displaced persons camps in Somalia: A 2 × 2 factorial cluster-randomised controlled trial
Source: PLoS Med. 2023 Feb 27;20(2):e1004180. doi: 10.1371/journal.pmed.1004180 (PMC9970051; doi:10.1371/journal.pmed.1004180)
Supplement: S7 Table — (DOCX) [file pmed.1004180.s008.docx]

**Table A7.** Unadjusted primary outcome indicators at baseline, midline, and endline, in the mHealth vs control comparison.

|  | Baseline (R1, Jan-Feb 2019) | | | | Midline (R3, Jun 2019) | | | | Endline (R9, Dec 2019) | | | |
| --- | --- | --- | --- | --- | --- | --- | --- | --- | --- | --- | --- | --- |
|  | Control | | mHealth | | Control | | mHealth | | Control | | mHealth | |
|  | n | % or  mean | n | % or  mean | n | % or  mean | n | % or  mean | n | % or  mean | n | % or  mean |
| Maternal/caregiver  Knowledge^1^ (score) | 373 | 11.3 | 407 | 12.3 | 357 | 13.9 | 388 | 14.8 | 344 | 13.8 | 371 | 15.0 |
| Measles  Vaccination^2^ (%) | 482 | 45.0 | 544 | 55.3 | 481 | 75.1 | 558 | 72.2 | 453 | 79.0 | 530 | 74.7 |
| Pentavalent series  vaccination^3^ (%) | 136 | 47.8 | 169 | 55.0 | 140 | 67.1 | 163 | 73.0 | 122 | 64.8 | 144 | 74.3 |
| Timely EPI  vaccination^4^(%) | 587 | 18.2 | 657 | 24.1 | 598 | 21.2 | 662 | 22.1 | 615 | 18.2 | 688 | 15.7 |
| Child dietary  Diversity^5^ (score) | 204 | 3.01 | 249 | 2.88 | 205 | 3.40 | 232 | 3.81 | 181 | 3.61 | 201 | 4.05 |

^1^ We assessed this outcome in mothers/caregivers.

^2^ We assessed this outcome in children aged 9-59 months.

^3^ We assessed this outcome in children aged 12-23 months.

^4^ We assessed this outcome in children aged 0-59 months. EPI, Expanded Programme on Immunization.

^5^ We assessed this outcome in children aged 6-23 months.
