# Supplementary material for: Evaluation of conditional cash transfers and mHealth audio messaging in reduction of risk factors for childhood malnutrition in internally displaced persons camps in Somalia: A 2 × 2 factorial cluster-randomised controlled trial
Source: PLoS Med. 2023 Feb 27;20(2):e1004180. doi: 10.1371/journal.pmed.1004180 (PMC9970051; doi:10.1371/journal.pmed.1004180)
Supplement: S8 Table — (DOCX) [file pmed.1004180.s009.docx]

**Table A8.** Combined effects of the conditional cash transfer (CCT) and mHealth interventions on primary outcomes

| **Intervention arm** | **Midline** | | | **Endline** | | |
| --- | --- | --- | --- | --- | --- | --- |
|  | **Odds Ratio^1^** | **95% CI** | ***P*** | **Odds Ratio** | **95% CI** | ***P*** |
| ***Measles Vaccination*** |  |  |  |  |  |  |
| No intervention | reference | - | - | reference | - | - |
| CCT | 18.41 | 5.35, 63.29 | <0.001 | 30.46 | 8.89, 104.31 | <0.001 |
| mHealth | 0.61 | 0.23, 1.60 | 0.317 | 0.42 | 0.12, 1.52 | 0.185 |
| CCT and mHealth | 4.71 | 1.58, 14.01 | 0.005 | 11.07 | 3.74, 32.76 | <0.001 |
| ***Pentavalent vaccination*** | | | | | | |
| No intervention | reference | - | - | reference | - | - |
| CCT | 5.05 | 2.05, 12.45 | 0.000 | 19.79 | 4.01, 97.54 | <0.001 |
| mHealth | 0.49 | 0.17, 1.43 | 0.191 | 0.60 | 0.16, 2.25 | 0.447 |
| CCT and mHealth | 6.28 | 1.15, 34.37 | 0.034 | 31.89 | 6.18, 164.64 | <0.001 |
| ***Timely vaccination*** |  |  |  |  |  |  |
| No intervention | reference | - | - | reference | - | - |
| CCT | 0.86 | 0.12, 5.98 | 0.881 | 0.82 | 0.21, 3.19 | 0.779 |
| mHealth | 0.49 | 0.09, 2.55 | 0.394 | 0.66 | 0.26, 1.67 | 0.381 |
| CCT and mHealth | 0.83 | 0.20, 3.53 | 0.804 | 0.22 | 0.06, 0.73 | 0.014 |
| ***Maternal/caregiver knowledge*** | | | | | | |
| No intervention | reference | - | - | reference | - | - |
| CCT | 0.47 | 0.05, 4.88 | 0.529 | 0.39 | 0.07, 2.16 | 0.282 |
| mHealth | 0.54 | 0.06, 5.14 | 0.591 | 1.06 | 0.12, 9.48 | 0.960 |
| CCT and mHealth | 1.10 | 0.04, 29.60 | 0.954 | 0.69 | 0.03, 15.40 | 0.815 |
| ***Child diet diversity*** |  |  |  |  |  |  |
| No intervention | reference | - | - | reference | - | - |
| CCT | 0.68 | 0.22, 2.10 | 0.505 | 0.56 | 0.27, 1.15 | 0.113 |
| mHealth | 1.71 | 0.71, 4.15 | 0.234 | 1.24 | 0.39, 3.89 | 0.716 |
| CCT and mHealth | 1.63 | 0.51, 5.25 | 0.406 | 2.66 | 1.41, 5.03 | 0.002 |

^1^ Odds ratios are from mixed effects logistic regression adjusted for baseline values
