# Supplementary material for: Evaluation of conditional cash transfers and mHealth audio messaging in reduction of risk factors for childhood malnutrition in internally displaced persons camps in Somalia: A 2 × 2 factorial cluster-randomised controlled trial
Source: PLoS Med. 2023 Feb 27;20(2):e1004180. doi: 10.1371/journal.pmed.1004180 (PMC9970051; doi:10.1371/journal.pmed.1004180)
Supplement: S1 Study Protocol — (DOC) [file pmed.1004180.s010.doc]

**Cash for Improved Nutrition in Somalia (CINS)**

**Study Protocol**

**Version Date: 12/11/2018**

**Project funded by:**

**Office of U.S. Foreign Disaster Assistance**

**USAID/DCHA/OFDA Annual Program Statement (APS) No. APS-OFDA-17-000002 for Leveraging Academic Partnerships in Operational Humanitarian Environments**

**Acronyms and Abbreviations**

AIM-WG Assessment and Information Management Working Group

AMISOM African Union Mission in Somalia

ARI Acute Respiratory Infections

AWD Acute Watery Diarrhoea

BCC Behaviour Change Communication

CBI Cash Based Intervention

CDR Crude Death Rate

CHWs Community Health Workers

CINS Cash for Improved Nutrition in Somalia

CTP Cash Transfer Program

CWW Concern Worldwide

FDG Focus Group Discussion

FSNAU Food Security and Nutrition Analysis Unit

GAM Global Acute Malnutrition

HH House Holds

IDPs Internally Displaced Persons

IYCN Infant and Young Child Nutrition

ISRCTN International Standard Randomised Controlled Trial Number

MCHN Maternal and Child Health and Nutrition

MEB Monthly Expenditure Basket

MoH Ministry of Health

MUAC Mid-Upper Arm Circumference

NIP Northern Inland Pastoral

NMS Nutrition and Mortality Monitoring System

OCHA Office for the Coordination of Humanitarian Affairs

ODK Open Data Kit

OFDA Office of US Foreign Disaster Assistance

REFANI Research on Food Assistance for Nutritional Impact

REFANI-S Research on Food Assistance for Nutritional Impact - Somalia

SAM Severe Acute Malnutrition

U5DR Under-Five Death Rate

UCL University College London

UCT Unconditional cash transfer

Contents

[Introduction 4](#__RefHeading___Toc529861990)

[Study Goal 5](#__RefHeading___Toc529861991)

[Study Objective 5](#__RefHeading___Toc529861992)

[Primary Research Question 5](#__RefHeading___Toc529861993)

[Study Setting 5](#__RefHeading___Toc529861994)

[Interventions 6](#__RefHeading___Toc529861995)

[Cash Transfer Intervention 6](#__RefHeading___Toc529861996)

[Behaviour Change Communication (BCC) Intervention 8](#__RefHeading___Toc529861997)

[Study Design 9](#__RefHeading___Toc529861998)

[Outcome measures 10](#__RefHeading___Toc529861999)

[Sample Size Calculations 10](#__RefHeading___Toc529862000)

[Quantitative Data collection 11](#__RefHeading___Toc529862001)

[Qualitative data collection 12](#__RefHeading___Toc529862002)

[Data Management 13](#__RefHeading___Toc529862003)

[Data Analysis 13](#__RefHeading___Toc529862004)

[Staff Training and Study Management 13](#__RefHeading___Toc529862005)

[Risk Management 14](#__RefHeading___Toc529862006)

[Ethical Review 15](#__RefHeading___Toc529862007)

[Coordination with Stakeholders and Results Dissemination 15](#__RefHeading___Toc529862008)

**Figures**

[Figure 1 Flow Diagram of Cash Registration System for Participants in the Conditional Cash Arm 7](#__RefHeading___Toc529861980)

[Figure 2 Flow Diagram of Audio Messaging for Participants in the BCC Arm 9](#__RefHeading___Toc529861981)

[Figure 3 Schematic of data collection schedule 11](#__RefHeading___Toc529861982)

# Introduction

It is estimated that in low and middle-income countries acute malnutrition affects about 52 million children aged less than 5 years [1]. While acute malnutrition is closely associated with poverty, its prevalence and severity is often greater in conflict and disaster affected areas [2]. Acute malnutrition is a leading cause of childhood mortality, accounting for 11.5% of total deaths, and contributes significantly to the global disease burden [1, 3].

Somalia has been affected by decades of political turmoil, protracted armed conflict, and intermittent natural disasters. These factors have contributed to one of the highest global prevalences of acute child malnutrition (17.4% as of April-June 2017) [4]. Conflict has disrupted not only regional agriculture and trade, but also health services and humanitarian access, increasing food insecurity, displacement and mortality, especially during periods of crisis [5]. In 2017-2018, it is estimated that 1.2 million children in Somalia will be acutely malnourished, of which over 230,000 will be severely malnourished [4]. The group most affected by food insecurity and acute malnutrition are internally displaced persons (IDP), who often live in camps in peri-urban areas and lack access to essential services [4].

Various humanitarian interventions are commonly used for the management of acute malnutrition. Cash-based interventions (CBI) have recently gained popularity compared to conventional food-based interventions [6]. CBI aim to improve the beneficiaries’ ability to acquire food and/or other needs; and they often have additional multi-sectoral objectives, such as enabling livelihood investments and can improve health outcomes [7, 8]. Furthermore, CBI are perceived as a cost-effective nutritional intervention that improves beneficiary satisfaction, and that have a positive impact on local economies.

CBI were first implemented at scale in Somalia in 2011 to respond to the famine crisis and, given the existence of functioning markets in this context and the lack of humanitarian access to southern Somalia, were seen as an essential approach for providing food assistance [9]. However, evidence of nutritional impact of CBI in this context is lacking.

The Research on Food Assistance for Nutritional Impact-Somalia (REFANI-S) consortium conducted a trial of an emergency unconditional cash transfer (UCT) program in the Afgooye Corridor, Mogadishu, Somalia in 2016 [10]. The study found that a combined UCT, non-food item kit, and free piped water increased food security, diet diversity, and reduced negative coping strategies in recipient households. However, longitudinal community surveillance showed no evidence of the UCT reducing the incidence or prevalence of malnutrition (MUAC <12.5cm or oedema). The findings of this research were consistent with other studies of CBI and nutrition and health that indicate the variable effect of CBI on nutritional indicators.[11-15]

Nonetheless, CBI are perceived to be cost effective, improve the beneficiaries’ satisfaction and impact positively on local economies as long as they are designed and implemented appropriately in a suitable context. Therefore, more information on the use and effectiveness of CTPs in the management of health and acute malnutrition during emergencies is critical, so as to understand under what circumstances they can be effective and what additional inputs may be required.

Behaviour change communication (BCC) interventions have the potential to change behaviours and improve health and nutrition in some contexts [16-18]. Delivery of BCC via mHealth[[1]](#footnote-2) approaches have been shown to be effective although the evidence suggests that the application of behaviour change theory has been sub-optimal [19]. There is some evidence that mobile phone messaging reminders for attendance at healthcare appointments can be effective at improving attendance and reducing missed appointments [20]. A 2015 meta-analysis found that mobile phone SMS messages had a small, positive, effect on a broad range of healthy behaviours and recently the potential for mHealth interventions to impact on food choice has also been demonstrated [21-23]. mHealth intervention in low income settings is a rapidly evolving field and its use in humanitarian contexts is an emerging area of great interest and public health potential. Combining mHealth interventions with cash or voucher transfers to achieve health objectives is an under researched topic although studies have assessed the feasibility of the approach [24]. A 2018 review called for more rigorous evaluation of mHealth interventions targeting maternal and child health [25].

This research project will develop mHealth BCC approaches to address key infant and young child feeding (IYCF) behaviours, caring practices, WASH practices, and health seeking behaviour in an emergency affected IDP population. A mHealth BCC component, if effective, could be a cost-efficient component to add to cash transfers that reach populations in insecure/inaccessible areas, as well as populations on the move. The interventions, if effective, have a high feasibility of being scaled up in Somalia and elsewhere around the world.

Using a randomised cluster-controlled 2x2 design, this study aims to assess whether a conditional cash transfer combined with mHealth BCC audio messaging, delivered through mobile phones, can improve vaccination coverage, child dietary diversity, breastfeeding practice and thereby reduce the risk of acute malnutrition in children aged 0-59 months, living in IDP camps near Mogadishu, Somalia.

# Study Goal

To ensure more effective humanitarian interventions by strengthening the evidence base on the use of cash transfers to prevent undernutrition in contexts of protracted displacement.

# Study Objective

To assess the impact of cash transfers, delivered together with behaviour change communication and conditional upon attending child health visits, on reducing the risk of acute malnutrition among children 6-59 months.

# Primary Research Question

Can cash transfers coupled with mHealth BCC and conditional upon attending a child health visit contribute to a reduction in risk factors for acute malnutrition in children, aged 6-59 months, in a crisis context characterized by significant population displacement?

# Study Setting

The research will take place in the Afgooye Corridor, an area close to urban Mogadishu. The Afgoye Corridor contains the largest IDP settlements in Mogadishu, a result of multiple protracted crises in the country. These settlements have become a safe-haven for IDPs fleeing conflicts and disasters in the regions around Mogadishu, including Lower and Middle Shabelle, Bay and Bakool regions. The IDPs subsist almost entirely on humanitarian assistance from relief agencies and from casual labor whenever such opportunities become available.

Concern has worked in Somalia, including the urban and peri-urban areas of Mogadishu since 1986. Concern has have strong security procedures as well as good relationships with local government and community leaders that have allowed safe and continuous operation during periods of high insecurity. While we have confidence that we will be able to implement the program in the planned areas, Concern also has a high capacity to implement in other areas of Somalia as well. We are flexible to work in different areas of Mogadishu based on security. We also have an operational presence in Baidoa where we also were initially planning to implement the research. In case of serious increased insecurity in Mogadishu, the project could shifted to Baidoa. The UCL research team has been working in Somalia since 2009, and the UCL Co-Investigator/Study Coordinator has worked extensively on research projects and programs within Somalia over the past 5 years.

We consider that the research question we are seeking to answer is best addressed in a complex emergency situation with high levels of acute malnutrition, and the proposed study site provides a very useful location in which this can be done.

# Interventions

Two interventions will be tested: unconditional vs. conditional cash and behaviour change vs. no behaviour change.

## Cash Transfer Intervention

Cash will be provided to households either as an unconditional or conditional cash transfer. All groups will receive cash transfers due to ethical reasons, as all households are considered to be suffering from extreme poverty and to be highly vulnerable. Randomization will be done at the cluster (camp) level. Households living in the selected camps will be listed and registered by the Concern cash distribution team.

The cash distribution will be done electronically each month and the specified amount will be sent to the registered SIM card of the beneficiary in US$. The cash transfers will be implemented by Hormuud Telecom via their EVC Plus money transfer service.[[2]](#footnote-3) Once the beneficiaries receive the cash transfer they can then use it to purchase items electronically or exchange for physical cash at a wide range of local shops. If they experience problems with use of the cash transfer they can contact the local Hormuud Telecom office for assistance. Problems can also be reported to the Concern beneficiary help line.

Cash transfers will follow the standard amounts set by the Somalia Cash Working Group based on a percentage of the Monthly Expenditure Basket (MEB). The cash transfers will be provided for 9 months. For the first 3-6 months the transfer amount provided will be that given during a humanitarian cash transfer programme; for the following 6 months the amount provided will be aligned with that given by social safety net (welfare) programmes.

The conditional cash transfer will require households with children, aged 0-59 months, to have first attended the local health clinic where they will receive a health screening, any necessary vaccinations, deworming, and vitamin A supplementation as required; and they will be issued with a child health record card. All the study camps have similar access to local health clinics. The health clinic visit will be verified by asking for the child health record cards during registration of the household for cash transfers.

Checks of child health card will be carried out monthly during the monthly monitoring household visits by CHWs. The check will monitor whether all children in the household 0-59 months are up to date on routine vaccination, vitamin A supplementation, and deworming.

The CCT and the UCT will be the same total amount per month, with equal amounts in all arms. Figure 1 shows participant flow for those in the study arm receiving conditional cash. Those receiving unconditional cash will not be advised to visit to the health clinic prior to cash distribution (although they are fully entitled to do so if they choose to) and they will not be asked to show a health record card for their children before being registered on the cash distribution list.

Figure 1 Flow Diagram of Cash Registration System for Participants in the Conditional Cash Arm


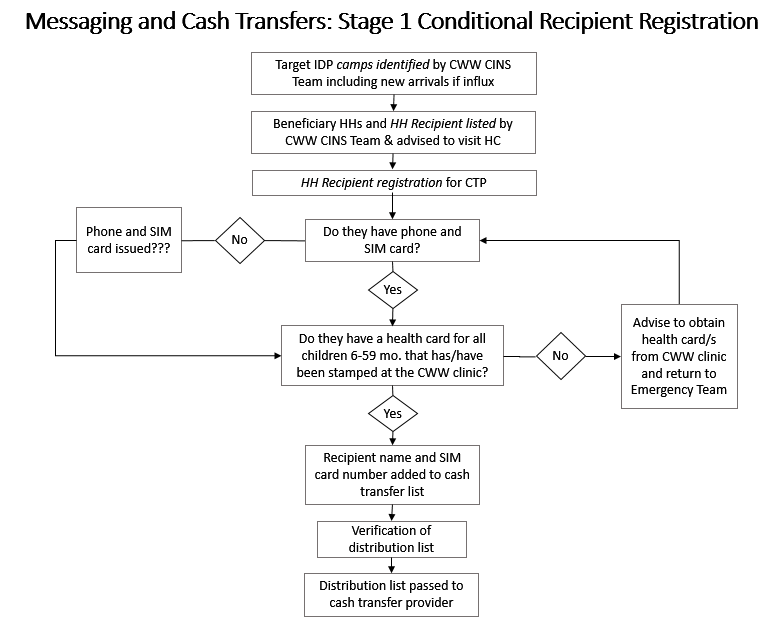


## Behaviour Change Communication (BCC) Intervention

All study arms will receive behaviour change communication (BCC) provided by CHW during their monthly household visits. The mHealth intervention arms will receive the additional mHealth BCC that will consist of audio messages transmitted to the recipient’s mobile phone. Provision of BCC messaging will be randomized among the groups at cluster (camp) level. The planned development strategy for the mHealth BCC messages has the following key points:

- - Messaging will be developed in collaboration with Shaqodoon and Media Inc., which are companies based in Somalia and are specialists in interactive voice response (IVR) technology and creative message development, respectively.
  - Currently, we plan to deliver 12 audio messages, with two messages focused on each of the 6 health and nutrition topics listed in Table 1 below. These topics were identified as major barriers to optimal nutrition in the REFANI research.
  - Messaging will be designed following a human centred design approach, will be culturally appropriate, and will be produced using local voice actors and musicians.
  - Message content will be tested to ensure it is compelling and effective. Different formats for message delivery will be tested, such as a 6-12 episode soap opera series, or standalone short/succinct messages.
  - The messages produced will be piloted with small groups of IDP living in the same area as the study population, and revised during several iterative rounds of development prior to general use.
  - Full consideration will be given to ensuring the messaging will be acceptable to all parties of the conflict. This will ensure that listening to the message does not put any beneficiaries at risk.

Table 1 List of Topics to be Covered in mHealth Messaging


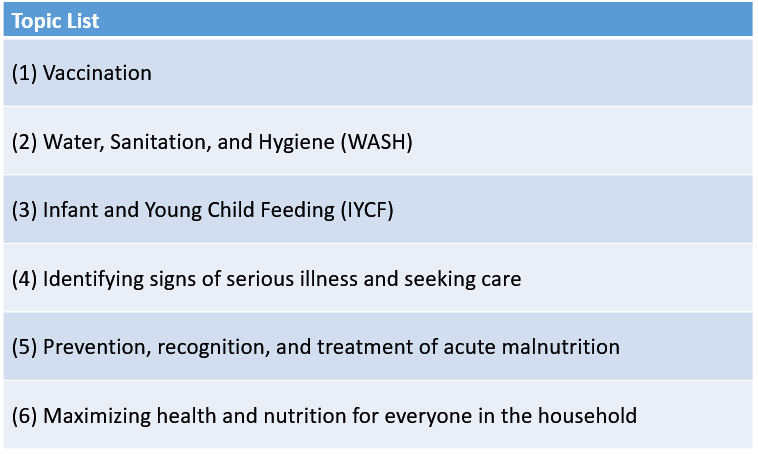


The developed audio messages will be delivered via a server, connected to the cellular network, to the registered mobile phone/SIM card of the participants in the BBC arm of the study. Figure 3 illustrates the messaging flow and associated data capture. Cash transfers are not conditional on listening to the audio messages but information on the number of messages listened to and the duration of the listening will be recorded.

Figure 2 Flow Diagram of Audio Messaging for Participants in the BCC Arm


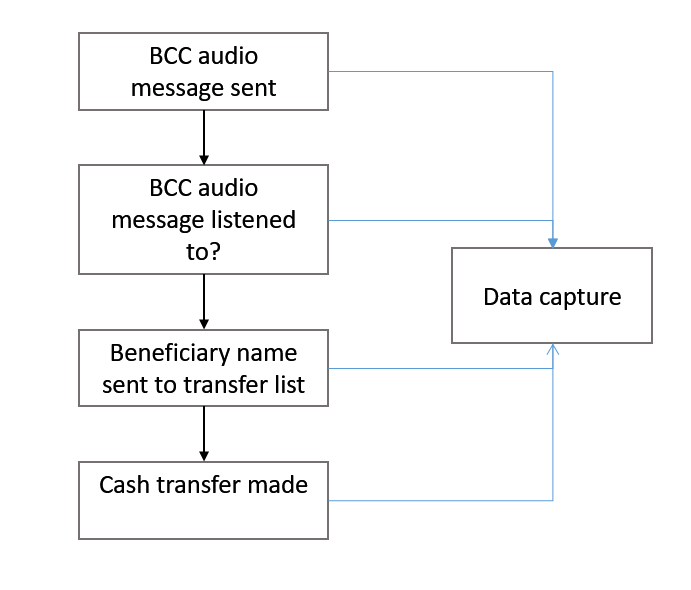


During their monthly household visits, CHW will support defaulter tracing, awareness raising, and community engagement in addition to the standard health and nutrition messaging work that is done routinely during household visits. These routine activities will be done equally in all study camps. To remove any potential bias between study arms due to differences in the quality of CHW services, CHW volunteers may be rotated between the camps in the different study arms. CHWs are volunteers but will be paid a monthly incentive

# Study Design

The research will take place in 20 IDP camps in the Afgooye Corridor, which are expected to contain an average population size of 65 children (aged 0-59 mo.) per camp. A 2x2 factorial design will be used with cluster randomization. A cluster will comprise one IDP camp. The study design is illustrated below.

Table 2 Study Design Summary

|  | Unconditional  Cash Transfer | Conditional  Cash Transfer |
| --- | --- | --- |
| No mHealth BCC | 5 camps | 5 camps |
| mHealth BCC | 5 camps | 5 camps |

All households within the 20 selected IDP camps will be invited to participate in the study so it is anticipated that a total of 1,300 children will be recruited. Measurements will be taken at monthly household visits, which will be done by community health workers, and continue during the course of the intervention. Children who exceed the age of 59 mo. during the course of the study will continue to be followed until the end of the follow-up period. Any new households that arrive in the IDP camps after the household recruitment has finished will not be invited to enrol.

Participant exclusion criteria:

1. Children with a disability that prevents taking weight or height measures

2. Children with a medical condition that prevents them eating a normal diet

3. Children confined to bed due to illness

4. Children living in a household in which the mother/carer is unable to respond to questions due to a speech or hearing impairment

This study design will allow us to address 2 main hypotheses:

(1) Humanitarian cash transfer conditionality improves health service utilization, increases vaccination coverage by 20%, and reduces morbidity in children aged 6-59 months.

This hypothesis will be tested by comparing the 10 clusters receiving unconditional cash with the 10 clusters receiving conditional cash transfers.

(2) A mHealth mobile phone BCC messaging intervention, linked to cash transfers, will improve health knowledge and nutrition related behaviours in a complex emergency context.

This hypothesis will be tested by comparing the 10 clusters who receive the mHealth intervention with those that do not. We will also test the secondary hypothesis that a combined conditional cash transfers and mHealth mobile phone BCC messaging intervention reduces the risk of acute malnutrition compared to those receiving unconditional cash transfers or no mHealth intervention.

# Outcome measures

- Measles vaccination coverage - % of children 9-<59 months of age who received measles vaccine
- EPI vaccination coverage - % of children 0-<59 months of age who received all vaccines required by the national vaccination protocols
- Child diet diversity score of children 6-<24 months of age
- Parental/caretakers knowledge of BCC health and nutrition topics

The secondary outcomes of interest are:

- Incidence of acute malnutrition - MUAC<12.5 cm or oedema among children 6-59 months
- Incidence of mortality among children 6-59 months
- Exclusive breastfeeding prevalence - % of infants 0-5 months who were exclusively breastfed during the last 24 hours ​
- Incidence of child morbidity
- Causes of death ascertained by Verbal Autopsy

# Sample Size Calculations

The sample size available to test for differences in vaccination coverage (hypothesis 1) was assessed using clustersampsi in Stata v14. Based on current measles vaccination coverage data, collected during the REFANI trial, we expect to see a baseline coverage of around 64%. With 10 clusters (camps) per arm and an average population of 65 children (aged 0-59 months) per cluster, and allowing for an alpha risk of 0.05, a power of 0.8, and an ICC of 0.06, we will be able to detect a minimum increase of 18 percentage points. This minimum detectable difference lies below our hypothesized effect size of 20 percentage points.

To calculate a sample size to test hypothesis 2 - the effect of mHealth messaging on health knowledge and nutrition behaviours - we assumed that there will be no interaction between the mHealth and conditionality interventions. Under this assumption we assessed the utility of the available sample size to detect a difference in children’s diet diversity score between the clusters receiving the mHealth intervention and those that won’t. Based on data from the REFANI study we expect to see a baseline child diet diversity score of 3 and a SD of 2. With 10 clusters (camps) per arm and an average population of 65 children (aged 0-59 months) per cluster, and allowing for an alpha risk of 0.05, a power of 0.8, and an ICC of 0.01, we will be able to detect a minimum increase of 0.42 in the diet diversity score. These calculations confirm that the study design is well powered to detect differences in our primary outcomes.

# Quantitative Data collection

Quantitative data will be collected using questionnaires translated into the local Somali language, and addressed to the primary carer of the child included in the study. A household surveillance system will be set up to collect anthropometric data that will be used to evaluate whether the intervention changes the primary and secondary outcomes listed above. Data collection will be done monthly at the household level by a team of CHW, closely supervised by Concern Nutrition Supervisors and the Study Coordinator. The questionnaires will include the data collection listed below but note that not all variables will be collected each month. Figure 4 provides a schematic to explain the data collection plan for each month of the study.

Separate household, child (0-59 month), and Mother/questionnaires will be used.

(a) Household questionnaire: The data collected will include: camp name, HH ID, data of arrival, age, sex, region of origin, date of interview, reduced coping strategies index, household food insecurity access scale (HFIAS) score, food consumption score (FCS).

(b) Child (0-59 months) questionnaire: HH ID, personal ID, vaccination status, 7 day morbidity recall, 24 hour dietary recall, mid upper arm circumference (MUAC), and nutritional oedema.

(c) Mother/carer: HH ID, personal ID, age, parity, region of origin, clan, health and nutrition knowledge, diet diversity score

Those identified as malnourished or sick will be referred to local nutrition or health centres for treatment.

Figure 3 Schematic of data collection schedule

Full data collection will be undertaken at the time points marked in blue while a reduced questionnaire will be used at the other monthly data collection time points. This will enable effective monitoring of changes during the intervention period while reducing the burden on the participants and data collection team.


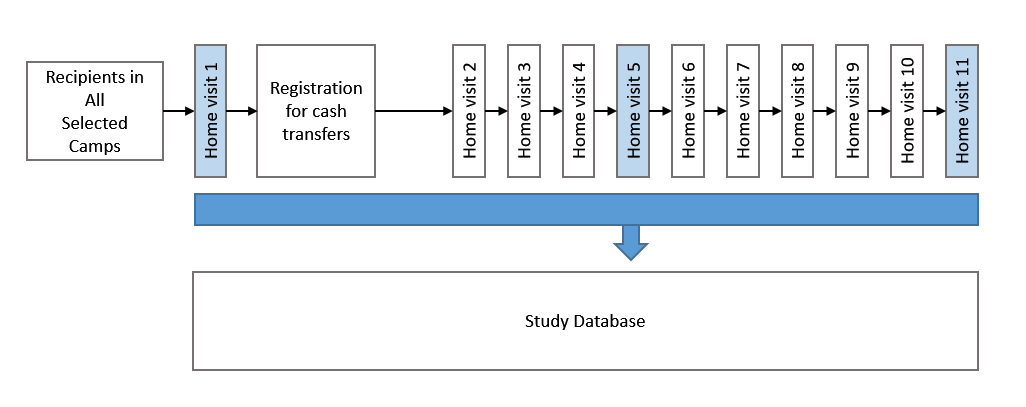


In the Somalia context, membership of clan and sub-clans is an important cultural and political factor. Clan membership is thought to influence vulnerability during crises and we will explore this hypothesis using the data we will collect on clan membership from all participants.

Verbal Autopsies will be conducted to determine the cause of death of any children who die during the study. When deaths are recorded by the study surveillance system, an interview team will conduct a household visit after an appropriate suitable period has elapsed. VA is the process of interviewing close caregivers of the deceased about symptoms and the circumstances of death. It has been widely used in research and over the past decade the WHO and partners have developed shorter, standardized survey tools and automated interpretation methods which can overcome barriers to their use in humanitarian contexts. It is now possible to administer a VA within about 20 minutes and immediately identify probable causes of death.

Trained fieldworkers will visit the next-of-kin in their residence within 4-8 weeks of notification of a death. Fieldworkers will administer the WHO 2016 VA questionnaire.[17] This questionnaire is comprised of a series of yes/no/don’t know questions relating to the signs, symptoms, circumstances and care-seeking (collectively called ‘indicators’) leading to death. Interviews will be conducted in Somali and data will be captured using a mobile phone with automated skip patterns and internal data checks.

VA data will be uploaded to a local database daily and run through InterVA software (www.interva.net). For every death, InterVA will identify multiple possible causes of death, each with an assigned likelihood. When aggregated, with individual deaths being split between multiple causes according to their likelihood weighting, these data give a representation of the cause distribution at the population level. The method has been applied in numerous settings and compares favourably to alternative means of identifying cause of death from VA with the added advantages of better timeliness, lower cost, and improved reliability.

# Qualitative data collection

The study will have a strong qualitative component. The qualitative data will be used to help inform the design of the intervention during the piloting stage (see above) and to understand the context of the study, the fidelity of the intervention implementation, and any unexpected outcomes of the interventions. Data will be collected using Interviews, focus group discussions (FDG), and observation notes. FGD and interviews will be recorded using handheld tape recorders. The qualitative data collection will be undertaken by a team of two data collectors, who have been trained in qualitative methods, and a supervisor.

Data collection will be carried out by mixed gender teams and qualitative research will be conducted by researchers of the appropriate gender to engender trust and openness in the different respondent groups. CHWs will be trained on culturally sensitive mobilization skills that help them understand how to work in communities during mobilization, research, and intervention activities. The time taken to conduct interviews will be minimized to reduce the opportunity costs for the household, and minimize the use of the respondent’s time. CHWs will also ensure all children are included in the surveillance system independent of whether they have an impairment (as children with disability are sometimes missed).

# Data Management

Data will, preferably, be collected on mobile digital devices, such as Samsung smart phones, using Open Data Kit (ODK) based software. Data will be uploaded to Ona Data servers as and when network connections are available. The uploaded data will be downloaded in standard.csv file format and imported into Stata v14 statistical analysis software. Relational databases will be constructed and data cleaning performed using standard techniques. Anthropometric indices will be calculated using WHO macros.

# Data Analysis

Data analysis will be conducted using Stata v14 software. Difference-in-difference analysis will be used to describe differences between arms in hypothesised mediating variables such as health and nutrition knowledge. Linear and logistic regression models will be used to test the main hypotheses while adjusting for covariates that are found to be associated with outcome measures and may act as potential confounders on the causal pathway.

Primary analysis will be conducted based on intention to treat. Secondary analysis will use monitoring and adherence data to assess for any dose response effect associated with the BCC or cash interventions.

Qualitative data will be transcribed in Somali, translated into English, and analysed using the framework method for thematic analysis. Analysis will be conducted using Nvivo qualitative data analysis Software (QSR International Pty Ltd. 2012. Version 11).

# Staff Training and Study Management

UCL will conduct face to face training of the study staff. The training locations used previously have been in either Mogadishu, Nairobi or Hargeisa, depending on the people involved and the security conditions pertaining at the time. Standard team training methods will be used including sessions on background theory, practical training on anthropometric measurements, role playing interview procedures, and piloting. Standardization tests will be performed to assess the participant’s ability to take accurate and precise measurements, and to record and enter data reliably. Participants who fail to achieve minimum standards will receive further training and/or not be deployed.

Community Health Workers (CHWs) will be selected from within the study communities and will receive training on anthropometric measurement, identification and referral of malnourished children and other people requiring medical attention, and referring young children for vaccinations. CHWs will also be trained on identification and referral of children with suspected malaria, diarrhoea, and pneumonia. CHWs will be recruited after administering a test and a face to face interview with Concern Health and Nutrition staff members in partnership with local authorities.

The Study Coordinator will provide regular on the ground support during data collection and, when this is not possible, will provide regular communication and support via internet and phone communication. The Study Coordinator and the Field Coordinator will supervise all field-teams. All the study team except the Study Coordinator will be local staff and will be based in the Mogadishu area, minimizing the risk of staff shortages due to road or airport closures.

# Risk Management

Currently, Mogadishu is considered relatively secure, although there are intermittent attacks by armed opposition groups against government officials and military forces allied with the government. There are also incidents of kidnapping for ransom and other criminal activities.

It is anticipated that Concern will have continued access to research areas in Mogadishu, with no serious escalation in conflict or deterioration in the political situation which would cause research areas to become inaccessible to either Concern or its partner staff. A critical assumption is that there will be no deterioration in the security situation which would make the presence of national or international staff untenable within the study area. We presume continued effective and consistent support from all stakeholders involved in this project.

Table 3 Risks and Contingencies/Alternative Strategies

| **Risk Factors** | **Contingencies/Alternative Strategies** |
| --- | --- |
| Inaccessibility of project  sites due to security concerns | A researcher of Somali origin from Kenya will oversee the research, closely supervising Somali national staff with strong capacity to undertake the research. Flexibility in approach is of paramount importance. Detailed options for re-scheduling, relocating, or modifying study activities in response to changes in the security conditions or other factors will be developed at the start of the project. Regularly review of security status with local authorities and institutions will be done and all teams on the ground will be kept well informed with security updates. |
| Lack of support/political goodwill by the community | All efforts will be made been made to align the research activities to the usual community household activity calendar/schedule whilst ensuring they will not be time-consuming. |
| Natural disaster (diseases, drought, pests, floods, etc. of unprecedented magnitude) | Deliberate efforts will be made to ensure that the project is complemented by other ongoing emergency interventions. In case of a large scale disaster, emergency funds from other donors will be mobilized. |
| Political stability at national level does not  provide enabling environment | A flexible implementation strategy has been designed. All relevant government officials at national and regional level will be kept well informed about the project through an inception meeting. In addition the community will be kept well informed of program. |
| Unnoticed and forceful eviction of research participants from IDP camps | Proper follow up and defaulter tracing mechanism will be employed to ensure that as many as possible participants complete the entire study duration. |
| Discontinuation of cash transfers which causes participants to seek humanitarian assistance in other areas. | Concern will work with donors, MOH and partners to conduct the study in areas most in need of CTP over the program period. |

During the previous REFANI-S study, Concern and UCL have developed robust approaches to ensure the quality of quantitative and qualitative data collection in a difficult security context where international researchers are generally not allowed full access and where many local staff have not had the opportunity to pursue advanced training. The HR strategy has been to recruit strong Somali nationals and invest heavily in their training and support, including UCL by researchers that provide regular training, mentoring and close supervision.

The Study Coordinator is from Kenya and is able to visit the study sites regularly and provide technical support to the teams.

In addition, the team on the ground are supported by the Concern Somalia Health and Nutrition Coordinator, Dublin based health, nutrition and research advisors, as well as the Somalia Programme Director, who has experience in research and program management. This approach has allowed Concern and UCL to build up a strong cadre of Somali research staff for quantitative and qualitative data collection. This team will be utilized during the CINS research and the same approach used, thereby building local capacity and ensuring a deep understanding of the local context.

# Ethical Review

Ethical approval has been obtained from the Ministry of Health, Mogadishu (Ref Number: MOH&HS/DGO/0993/Jun/2018) and is being sought from the Research Ethics Committee of University College London. The study will be registered with ISRCTN. Informed verbal consent will be obtained from camp leaders in all camps before the start of data collection. Informed consent, verbal and written, will be sought from caregivers at the household level following a detailed explanation of the study objectives and the data that will be collected. In addition, they will be informed about their right to withdraw from the study and that participation or withdrawal from the study will not affect their entitlement to humanitarian assistance. All data collection procedures will ensure the anonymity of the respondents. This includes the removal of any spatial data from datasets that could be used to identify the precise location of respondents.

# Coordination with Stakeholders and Results Dissemination

Sub-national and national cluster meetings as well as inter-cluster coordination meetings led by the MoH, UNICEF, and OCHA already exist as forums for information sharing. Sub-national Nutrition Cluster coordination meetings are very useful for information exchange which feeds into the National Nutrition Cluster assessment and response to the situation.

Concern is a member of the Nutrition Cluster’s Assessment and Information Management working group (AIM WG) which is mandated to vet and validate all studies done by nutrition partners and will therefore present the study protocol to the group for their validation before start of the study implementation. Concern will also keep the group informed about the study progress by periodically presenting the progress in the AIM WG’s monthly meetings.

Concern is also a member of both the Food Security Cluster and Cash WG which provide coordination and sector strategies. In these platforms, agencies share information regarding their areas of work and what activities they are doing in specific area. Concern will therefore keep both cluster and WG members informed about the research study area and interventions so that duplication of activities will be avoided.

**References**

1. Black RE, Victora CG, Walker SP, Bhutta ZA, Christian P, de Onis M, Ezzati M, Grantham-McGregor S, Katz J, Martorell R *et al*: Maternal and Child Nutrition 1 - Maternal and child undernutrition and overweight in low-income and middle-income countries. *The Lancet* 2013, 382(9890):427-451.

2. Kinyoki DK, Moloney GM, Uthman OA, Kandala N-B, Odundo EO, Noor AM, Berkley JA: Conflict in Somalia: impact on child undernutrition. *BMJ Global Health* 2017, 2(2).

3. Black RE, Allen LH, Bhutta ZA, Caulfield LE, de Onis M, Ezzati M, Mathers C, Rivera J, Maternal, Child Undernutrition Study G: Maternal and Child Undernutrition 1 - Maternal and child undernutrition: global and regional exposures and health consequences. *Lancet* 2008, 371(9608):243-260.

4. OCHA: 2018 Humanitarian Needs Overview: Somalia. In*.*; 2017.

5. Seal A, Checchi F, Balfour N, Nur A-RH, Jelle M: A weak health response is increasing the risk of excess mortality as food crisis worsens in Somalia. *Conflict and Health* 2017, 11(1):12.

6. Harvey P, Bailey S: Cash transfer programming in emergencies. In: *Good Practice Reviews.* Overseas Development Institute; 2011.

7. Sircar NR, Friedman EA: Financial security and public health: How basic income & cash transfers can promote health. *Glob Public Health* 2018:1-11.

8. Bailey S, Hedlund K: The impact of cash transfers on nutrition in emergency and transitional contexts: A review of evidence. In*.* London: Humanitarian Policy Group; 2012.

9. Hedlund K, Majid N, Maxwell D, Nicholson N: Final Evaluation of the Unconditional Cash and Voucher Response to the 2011–12 Crisis in Southern and Central Somalia. In*.*: Humanitarian Outcomes; 2012.

10. Jelle M, Grijalva-Eternod CS, Haghparast-Bidgoli H, King S, Cox CL, Skordis-Worrall J, Morrison J, Colbourn T, Fottrell E, Seal AJ: The REFANI-S study protocol: a non-randomised cluster controlled trial to assess the role of an unconditional cash transfer, a non-food item kit, and free piped water in reducing the risk of acute malnutrition among children aged 6–59 months living in camps for internally displaced persons in the Afgooye corridor, Somalia. *BMC Public Health* 2017, 17(1):632.

11. de Groot R, Palermo T, Handa S, Ragno LP, Peterman A: Cash Transfers and Child Nutrition: What We Know and What We Need to Know. In*.* Florence: UNICEF; 2015.

12. Pega F, Liu SY, Walter S, Lhachimi SK: Unconditional cash transfers for assistance in humanitarian disasters: effect on use of health services and health outcomes in low- and middle-income countries. *Cochrane Database of Systematic Reviews* 2015(9):11247-11247.

13. Langendorf C, Roederer T, de Pee S, Brown D, Doyon Sp, Mamaty A-A, TourÃ© LWM, Manzo M, Grais R: Preventing Acute Malnutrition among Young Children in Crises: A Prospective Intervention Study in Niger. *PLoS Medicine* 2014, 11(9):e1001714.

14. Grellety E, Babakazo P, Bangana A, Mwamba G, Lezama I, Zagre NM, Ategbo E-A: Effects of unconditional cash transfers on the outcome of treatment for severe acute malnutrition (SAM): a cluster-randomised trial in the Democratic Republic of the Congo. *BMC Med* 2017, 15(1):87.

15. Hagen-Zanker J, Bastagli F, Harman L, Barca V, Sturge G, Schmidt T: Understanding the impact of cash transfers: the evidence. In*.* London: ODI; 2016.

16. Paul KH, Muti M, Chasekwa B, Mbuya MNN, Madzima RC, Humphrey JH, Stoltzfus RJ: Complementary feeding messages that target cultural barriers enhance both the use of lipid-based nutrient supplements and underlying feeding practices to improve infant diets in rural Zimbabwe. *Maternal and Child Nutrition* 2012, 8(2):225-238.

17. Roy SK, Fuchs GJ, Mahmud Z, Ara G, Islam S, Shafique S, Akter SS, Chakraborty B: Intensive nutrition education with or without supplementary feeding improves the nutritional status of moderately-malnourished children in Bangladesh. *J Health PopulNutr* 2005, 23(4):320-330.

18. Bhutta ZA, Ahmed T, Black RE, Cousens S, Dewey K, Giugliani E, Haider BA, Kirkwood B, Morris SS, Sachdev HPS *et al*: Maternal and Child Undernutrition 3 - What works? Interventions for maternal and child undernutrition and survival. *Lancet* 2008, 371(9610):417-440.

19. Cho YM, Lee S, Islam SMS, Kim SY: Theories Applied to m-Health Interventions for Behavior Change in Low- and Middle-Income Countries: A Systematic Review. *Telemed e-Health*:15.

20. Gurol-Urganci I, de Jongh T, Vodopivec-Jamsek V, Atun R, Car J: Mobile phone messaging reminders for attendance at healthcare appointments. *The Cochrane database of systematic reviews* 2013(12):Cd007458.

21. Carfora V, Caso D, Conner M: Randomized controlled trial of a messaging intervention to increase fruit and vegetable intake in adolescents: Affective versus instrumental messages. *Br J Health Psychol* 2016, 21(4):937-955.

22. Carfora V, Caso D, Conner M: Randomised controlled trial of a text messaging intervention for reducing processed meat consumption: The mediating roles of anticipated regret and intention. *Appetite* 2017, 117:152-160.

23. Orr JA, King RJ: Mobile phone SMS messages can enhance healthy behaviour: a meta-analysis of randomised controlled trials. *Health Psychol Rev* 2015, 9(4):397-416.

24. Wakadha H, Chandir S, Were EV, Rubin A, Obor D, Levine OS, Gibson DG, Odhiambo F, Laserson KF, Feikin DR: The feasibility of using mobile-phone based SMS reminders and conditional cash transfers to improve timely immunization in rural Kenya. *Vaccine* 2013, 31(6):987-993.

25. Chen H, Chai YL, Dong L, Niu WY, Zhang PH: Effectiveness and Appropriateness of mHealth Interventions for Maternal and Child Health: Systematic Review. *JMIR mHealth uHealth* 2018, 6(1):12.

1. mHealth is a general term that describes the use of mobile phones and other technology in health promotion and medical care. The most common application of mHealth is the use of mobile phones and other digital devices to provide information to the target population and promote desired behaviours. [↑](#footnote-ref-2)
2. <https://www.hormuud.com/personal/services/evc-plus.aspx> [↑](#footnote-ref-3)
